# Cu(II) Stability and UV-Induced Electron Transfer in a Metal–Organic Hybrid: An EPR, DFT, and Crystallographic Characterization of Copper-Doped Zinc Creatininium Sulfate

**DOI:** 10.1021/acs.jpca.4c06133

**Published:** 2024-11-20

**Authors:** Michael J. Colaneri, Simon J. Teat, Jacqueline Vitali

**Affiliations:** † Department of Chemistry and Physics, State University of New York at Old Westbury, Old Westbury, New York 11568, United States; ‡ Lawrence Berkeley National Lab, 1 Cyclotron Road MS 15RO317, Berkeley, California 94720, United States; § Department of Physics and Department of Biological, Geological and Environmental Sciences, Cleveland State University, Cleveland, Ohio 44115, United States

## Abstract

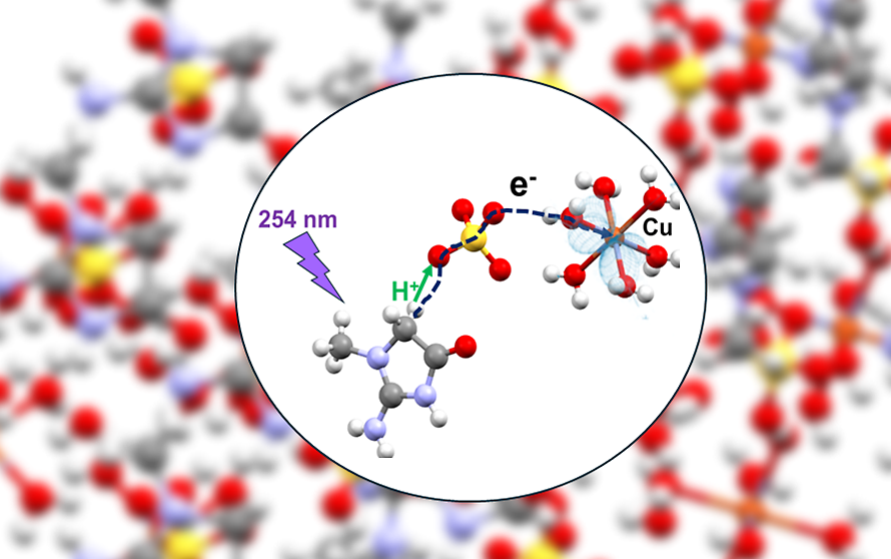

Single-crystal X-ray diffraction and electron paramagnetic resonance (EPR) spectroscopic experiments, complemented by quantum chemical DFT calculations, were carried out on the copper-doped metal–organic hybrid and Tutton salt analogue zinc creatininium sulfate to determine its crystal structure, to characterize the electronic structure of the doped Cu(II) binding site, and to propose a pathway for an excited-state, proton-coupled electron transfer (PCET) process in UV-exposed crystals. The crystal structure is isomorphous to that of cadmium creatininium sulfate, which has the transition ion, not in direct coordination with the creatinine, but forming a hexahydrate complex, which is bridged to a creatininium through an intervening sulfate ion. The EPR **g** (2.446, 2.112, 2.082) and copper hyperfine (**A**^**Cu**^: −327, −59.6, 10.8 MHz) tensor parameters are consistent with doped copper replacing host zinc in the metal–hexahydrate complex. These parameters are similar to those observed for copper hexahydrate in doped Tutton salt systems at low temperature, where the unpaired electron occupies mainly the copper 3*d*_*x*^2^–*y*^2^_ orbital. At room temperature in the Tutton systems, vibration couplings stemming from a dynamic Jahn–Teller effect cause tensor averaging which results in a reduction in their maximum *g*-tensor and hyperfine tensor values. However, like for the doped isomorphous Cd creatinine crystal, the Cu(II) EPR exhibits little, or no room temperature averaging compared to its low temperature pattern. Samples exposed to 254 nm UV light generate a carbon-centered free radical species, characterized by an isotropic g-tensor (*g* = 2.0029) and an alpha-proton hyperfine coupling (−24 −14 +4 G). These parameters identify it as a creatinine radical cation formed by the oxidative release of one of its C2 methylene hydrogens. DFT calculations confirm the unpaired electronic structures of both the Cu(II) site and free radical. The growth in radical concentration with an increase in the UV exposure time coincides with a decrease in the copper EPR signal, indicating a coupled light-induced oxidation reduction process. A comparison of the crystal structure with the EPR parameters and DFT results provides evidence for a UV-induced PCET.

## Introduction

Characterization of metal–organic hybrid systems has gained interest due to their wide-ranging potential, for example, in energy storage,^1,2^ as functional nonlinear optical^3^ and photovoltaic devices,^4,5^ catalytic agents,^6^ waste mediators,^7^ drug delivery,^8^ and many others.^9−11^ The reasons behind these unique applications include their layered or framework-caged metal and organic molecular structures that impart favorable electronic, topographical and physical characteristics.^1−11^ There has also been renewed interest in structurally related but inorganic double metal sulfate hexahydrates M^2+^2M^+^(SO_4_)_2_·6H_2_O known as Tutton salts, and more recently their mixed metal and doped counterparts, also due to their possible use in optical devices^12^ and thermal energy storage.^13^ The structural and electronic integrity of these materials under various environmental stressors is tantamount to their utility.^14−18^

Similar systems are also useful as biophysical probes that advance our understanding of the electronic structure, stability, and dynamics of metal cofactors in metalloproteins. Copper movement, plasticity, and translocations between active sites are inherently important for proper copper homeostasis^19,20^ and enzymatic activity.^21−24^ Previous focus in this lab has been on the characterization of metal “hopping” dynamics between sites related by crystallographic symmetry in biological models, as reported, for example, in Cu^2+^-doped, Zn-DL histidine,^25^ Cd-l-histidine,^26^ and Cd-DL histidine.^27^ This “hopping” process appears to be different from the ubiquitous dynamic Jahn–Teller effect long observed in copper-doped Tutton salts and other systems where the vibrational states of the metal hexahydrate complex couple the electronic states of Cu(II).^28−30^ Dynamic coupling causes a significant temperature-dependent averaging of the Cu(II) EPR parameters. However, in a recent temperature-dependent EPR investigations of the hexahydrate complex in the metal–organic Tutton analogue Cd creatininium sulfate (CdCrnS), there exists an almost complete lack of copper dynamics.^31^ Up until then, all known copper hexahydrate complexes in crystals showed significant dynamic behavior, and this exception was attributed to the small magnitude of the lattice electric field gradient along the copper–water bonds of the complex compared to that found in Tutton salt crystals.^32^ The notion that the lattice field gradient creates instability of the hexahydrate complex, thereby enhancing the dynamic process, was previously proposed based on comparisons with solid-state quantum chemical calculations.^33^

To continue this work, a combined crystallographic, DFT, and EPR investigation of Cu^2+^-doped Zn-creatininium (ZnCrnS) was carried out. X-ray crystallography shows that ZnCrnS is isomorphous to the Cd-creatininium sulfate structure. Our aim here is to assess the stability of the copper state in a second metal–organic Tutton analogue. During this investigation, an ultraviolet-induced reaction was observed that converts EPR-active Cu(II) to EPR-silent Cu(I) along with a concomitant formation of a free radical species. Only a few prior EPR studies have addressed the ultraviolet (UV) susceptibility of Cu(II) containing biological crystals with the formation of free radicals. In doped l-alanine crystals,^34^ UV exposure at 77 K has revealed some intermediate Cu^2+^ complexes during the process, while in glycine and methylglycine^35^ no intermediates were detected in the range 77–300 K. In glycylglycine, UV irradiation at 77 K again indicated the presence of short-lived, intermediate Cu(II) complexes during the free radical formation process.^36^ Due to the appearance of a tail in the UV absorption region of these copper containing crystals, the molecular damage products and copper reduction were concluded to occur within the first sphere via excitation of the ligand to copper charge-transfer absorption.^34^ A more recent, though preliminary, study has shown similar UV effects in irradiated powders of Cu(II)-doped histidine crystals.^37^ Given the limited number of these investigations, the current system provided an additional opportunity to study UV-induced molecular damage in a biological crystal and to assess how the presence of Cu^2+^ ions can mediate these processes.

### Experimental Section

Single crystals of copper-doped zinc creatininium sulfate were grown by slow evaporation of aqueous solutions containing creatinine and zinc sulfate, with 1–4% of copper sulfate. Light doping (<1%) of the copper ion into a diamagnetic host minimizes the complications resulting from interacting electron spins.^38−40^ The relatively high dope amounts employed here were necessary given the crystal size and spectrometer sensitivity, and did not appear to significantly affect the accuracy of the spectral parameters. The small prismatic crystals were pale blue. All compounds were of the highest purity obtained from Fisher. Some crystals showed weak EPR signals consistent with Mn^2+^ impurities, which were reduced but never fully eliminated by employing metal-free sulfuric acid and different water sources. A BIOMATE 3 UV–vis spectrometer was utilized to measure the transmission characteristics of a Newport U-340 UV bandpass filter.

### Crystallography

X-ray diffraction data were collected on a single crystal using a Bruker D8 diffractometer equipped with a PHOTONII detector and operating with a silicon 111 monochromator and synchrotron radiation of wavelength 0.7288 Å at 298(2) K and 100(2) K at beamline 12.2.1 of the Advanced Light Source at Lawrence Berkeley National Laboratory. The scans were shutterless. The same crystal was used for both data collections. The Bruker Apex 3 program was used for the data collection.^41^ Intensity data integrations, cell refinement, and data reduction were performed using the Bruker SAINT software package.^41^ Absorption correction was made with SADABS.^41,42^ Crystal data are given in Table 1.

**Table 1:** Crystal Data and Refinement of the 100 and 298 K Structures

	100 K	298 K
empirical formula^a^	C8 H32 Zn0.96 Cu0.04 N6 O18 S2	
formula weight^b^	629.81	
wavelength, Å	0.7288	
space group	*P*21/*n*	
unit cell dimensions	*a* = 6.3912(4) Å	*a* = 6.4433(15) Å
	*b* = 27.8593(18) Å	*b* = 28.051(7) Å
	*c* = 7.0916(5) Å	*c* = 7.1452(17) Å
	β = 111.237(2)°	β = 110.934(9)°
Volume	1176.94(14) Å^3^	1206.2(5) Å^3^
*Z*^c^	2	
Crystal size, mm^3^	0.30 × 0.25 × 0.20	
Density (calculated), Mg/m^3^	1.777	1.734
absorption coefficient, mm^–1^	1.398	1.365
absorption correction	semiempirical from equivalents	
maximum and minimum transmission	0.767 and 0.675	0.772 and 0.686
θ range for data collection	3.00 to 37.43°	2.98 to 30.02°
reflections collected	49,762	40,031
independent reflections	5714 [*R*(int) = 0.0549]	3264 [*R*(int) = 0.0604]
completeness to theta = 25.930°	99.8%	99.9%
refinement method	full-matrix least-squares on *F*^2^	
data/restraints/parameters	5714/0/217	3264/0/218
goodness-of-fit on *F*^2^	1.074	1.116
final *R* indices^d^ [*I* > 2sigma(*I*)]	*R*1 = 0.0256, *wR*2 = 0.0665	*R*1 = 0.0316, *wR*2 = 0.0
*R* indices^d^ (all data)	*R*1 = 0.0290, *wR*2 = 0.0681	*R*1 = 0.0354, *wR*2 = 0.0803
largest diff. peak and hole, *e* Å^–3^	0.516 and −0.467	0.623 and −0.551

a The empirical formula given corresponds to two asymmetric units related to each other by a crystallographic inversion center. Zn0.96/Cu0.04 is a mixed site at the inversion center. The asymmetric unit has one creatinine, one sulfate, three waters coordinated to the mixed Zn/Cu site at the inversion center and one additional water.

b The formula weight corresponds to the empirical formula.

c Z refers to the sum of two asymmetric units related by the crystallographic inversion center.

d *R*_1_ = Σ ||*F*_o_| – |*F*_c_||/Σ|*F*_o_|; *wR*_2_ = [Σ[*w* (*F*_o_^2^ – *F*_c_^2^)^2^]/Σ[*w* (*F*_o_^2^)^2^]]^1/2^.

The 298 K structure was determined with direct methods using SHELXT 2014/5^43^ and refined using SHELXL 2018.^43^ All non-hydrogen atoms were refined anisotropically. During refinement, the positions and displacement parameters of Zn and Cu were constrained to be the same at the inversion center. The occupancy of Cu was taken to be 0.02 and that of Zn 0.48 based on the fact that the solution was 4% Cu. Hydrogen atoms were obtained from electron density difference maps and allowed to refine freely except for the methyl group, where geometry constraints were used. The same procedure was adopted for the 100 K structure. Data collection and refinement details for both structures are given in Table 1. Further details, including atomic parameters, complete distances and angles, and hydrogen bonds are found in the Supporting Information. Superpositions were carried out with Olex2^44^ (https://www.olexsys.org). Ortep style figures were prepared with Platon.^45^

### EPR Spectroscopy

CW-EPR measurements were carried out using a Varian X-band E-109 spectrometer operating at 9.1 GHz and interfaced with a PC and gaussmeter as described in the past.^26,27,31,46,47^ DPPH (*g* = 2.0036) was used as a reference g value. An ESR900 Oxford Cryostat system employing liquid nitrogen was utilized for the low temperature EPR experiments. Crystals were mounted along the crystal **a**, **b**, and **c′** axes (where **c′** = **a** × **b**) with Duco cement on long Pasteur pipettes. These directions were used as a reference system for the EPR rotational measurements. The mounted samples were inserted into standard 3 × 4 mm EPR quartz tubes and attached to a Bruker goniometer for introduction into the TE102 cavity. This allows rotating the magnetic field direction in the crystal reference planes **ab**, **ac′**, and **bc′**, with an approximate uncertainty of about 2°. EPR spectra at 298 K were recorded digitally for magnetic-field orientations at 5° intervals in these three planes. Some crystals were manually crushed into powders and placed directly in the tubes for measurements. For the copper pattern, the **g** and copper hyperfine (**A**^**Cu**^) tensors were determined by a least-squares fit of the orientational spectral data to the spin Hamiltonian (eq 1) using methods described previously.^48^
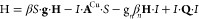
1with *S* = 1/2, *I* = 3/2 and the remaining terms defined in the usual way.^38^ In eq 1, the inclusion of a copper nuclear quadrupole tensor (**Q**) of (20, −10, −10 MHz),^49^ produced a better fit of the data. EasySpin^50^ was employed to fit and simulate powder EPR patterns. Experimental EPR analysis of the free radical pattern is described below.

### UV Irradiations and Free Radical Analysis

UV-irradiations were performed at room temperature with a UVP pen-ray finger Hg lamp on samples placed in a spot plate or on crystals mounted inside quartz EPR tubes. In some of these, a Newport UV bandpass filter (U-340) was utilized. Peakfit v4.12^51^ including its Levenberg–Marquardt (LMFit) fitting algorithm was used to integrate EPR patterns and fit center fields and line widths of the free radical doublet pattern to resolve proton hyperfine splitting values. Gnuplot v5.4^52^ routines were used to fit the proton hyperfine tensor components to the splitting orientation dependences in the three crystal reference planes according to the methods described later. EIGENG^53^ was used to compute the principal values and directions using these components.

### Quantum Chemical Calculations

Quantum chemical (QM) single-point energy and geometry optimization calculations were performed using Gaussian G16^54^ at the Ohio Supercomputer Center. ORCA 5.0.4^55,56^ was used to calculate the **g** and **A** tensors of ^63^Cu of the hexahydrate and ^1^H couplings of the free radical using methods implemented in the package. This included the contribution to the hyperfine tensors from the residual orbital motion of the unpaired electron. Theoretical properties were determined at the DFT level with various functionals and basis sets using chemical models detailed below. Atomic basis sets were used as supplied in the packages unless otherwise specified. Mercury^57^ was utilized to visualize and determine the atomic Cartesian coordinates of the models. Chimera^58^ produced structural figures and rendered the theoretical spin density surfaces.

## Results

### Description of the Structures

The structure is isomorphous to the Cd/Cu creatininium sulfate.^31^ Upon cooling, the unit cell shrinks slightly along **b** by 0.1917(72) Å and along **a** and **c** by 0.0521(16) Å and 0.0536(18) Å, respectively (Table 1). Matching of all parts of the asymmetric unit with OLEX2 gives rms of 0.147 Å for creatininium, 0.040 Å for the metal hexahydrate and 0.003 Å for the sulfate. As expected, the temperature factors are lower at 100 K.

**Figure 1:**
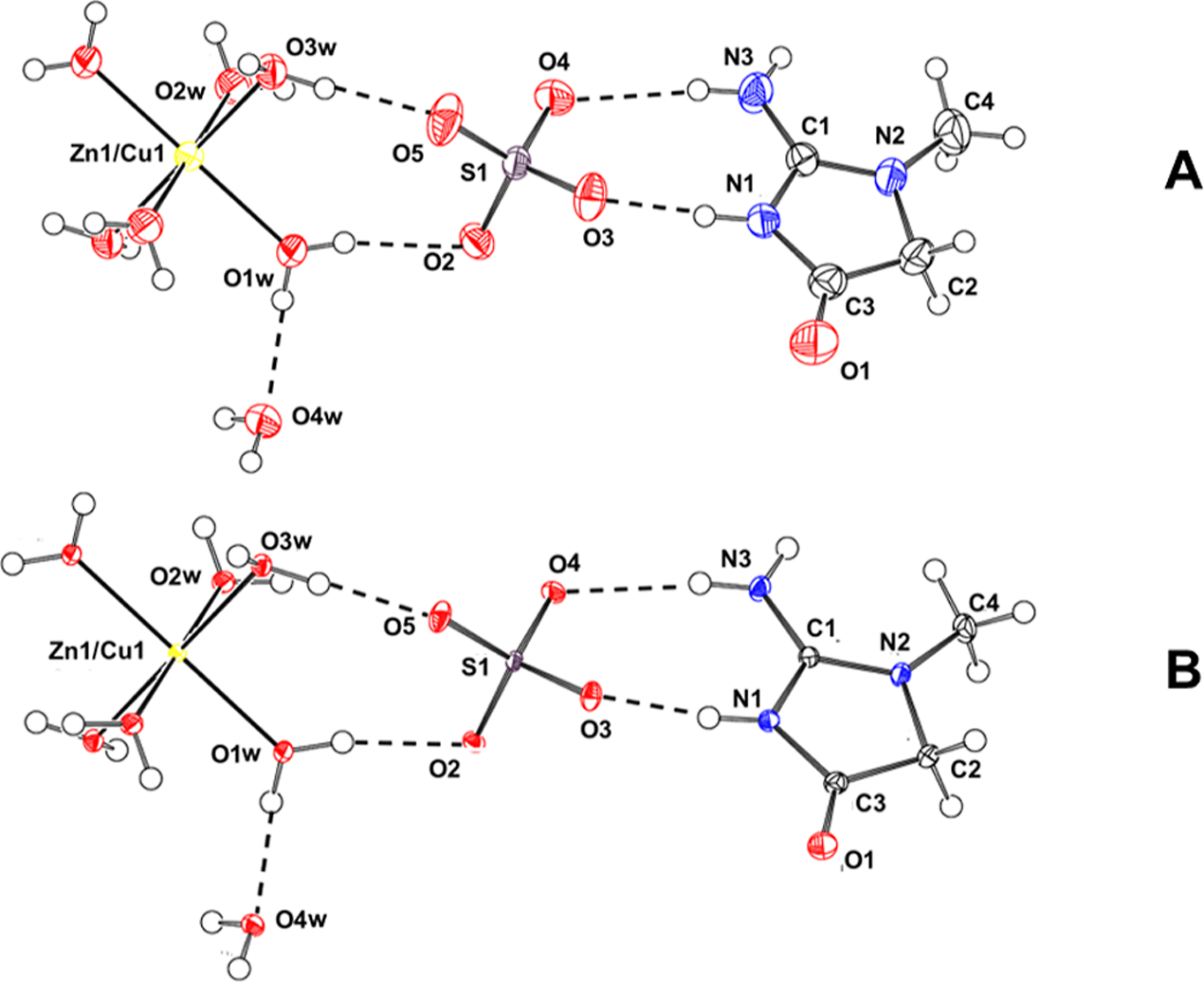
A view of the asymmetric unit at 298 K (A) and 100 K (B) with nomenclature for the non-hydrogen atoms. The asymmetric unit is augmented by three waters centrosymmetrically related to O1W, O2W and O3W and coordinated to the mixed Zn/Cu site at the inversion center. One may note the lower thermal motion of the 100 K structure. The figure was drawn with Platon.^45^

The asymmetric unit consists of a creatininium, a sulfate, 3 waters coordinated to a mixed Zn/Cu site at the inversion center and one solvation water (Figure 1). The Zn/Cu site is coordinated by six waters, three from the asymmetric unit and three from its centrosymmetrically related mate. The asymmetric unit shown in Figure 1 is expanded by symmetry to include the coordination of the Zn/Cu site at the inversion center. The Zn/Cu site adopts a slightly distorted octahedral coordination geometry. Bond lengths and angles are presented in Tables S3 and S9 for the 298 and 100 K structures, respectively. This coordination of the metal site by six waters is similar to that seen in the metal hexahydrate complexes of Tutton’s salts^59−68^ and other structural analogs of them with organic cations.^31,69−73^ The Zn/Cu-OW bonds are in the range 2.0532(12)–2.1480(11) Å at 298 K and 2.0502(6)–2.1330(6) Å at 100 K. The cis OW-Zn/Cu-OW angles range from 91.15(5) to 92.51(5)° at 298 K and from 91.22(3) to 92.65(3)° at 100 K. The trans OW-Zn/Cu-OW angles are 180° by the crystallographic inversion center. The average Zn-OW distances of 2.093(49) Å at 298 K and 2.086(43) Å at 100 K are similar to the metal hexahydrates of Zn tutton salts^61−68^ and, as expected, shorter than the average values of 2.269(45) Å at 298 K and 2.269(41) Å at 100 K of Cd-OW in cadmium creatininium sulfate.^31^ Likewise, the cis OW-Zn/Cu-OW angles are similar to the Zinc Tutton salts (e.g., in zinc potassium sulfate hexahydrate at 293 K, the cis angles range from 90.01 to 91.38°)^68^ and therefore display a comparable distortion from octahedron. The copper hexahydrates in these systems should therefore be formally characterized as pseudo-Jahn–Teller complexes.^33^ The creatininium ring is nearly planar with a rmsd of 0.005 Å at 298 K and 0.007 Å at 100 K. Bond distances and angles are comparable to other creatininium ions.^31,74−78^ Hydrogen bonds are given in Tables S6 and S12. The structure is held together by a network of hydrogen bonds and stacking interactions in a very similar manner to Cd/Cu creatininium sulfate.^31^ As in the Cd/Zn structure, one of the C2 hydrogens (H2A) hydrogen bonds to a neighboring sulfate O3 atom at (*x* – 1/2, −*y* + 3/2, *z* + 1/2). This hydrogen bond is involved in the stabilization of the free radical as discussed below.

### Copper EPR Analysis

**Figure 2:**
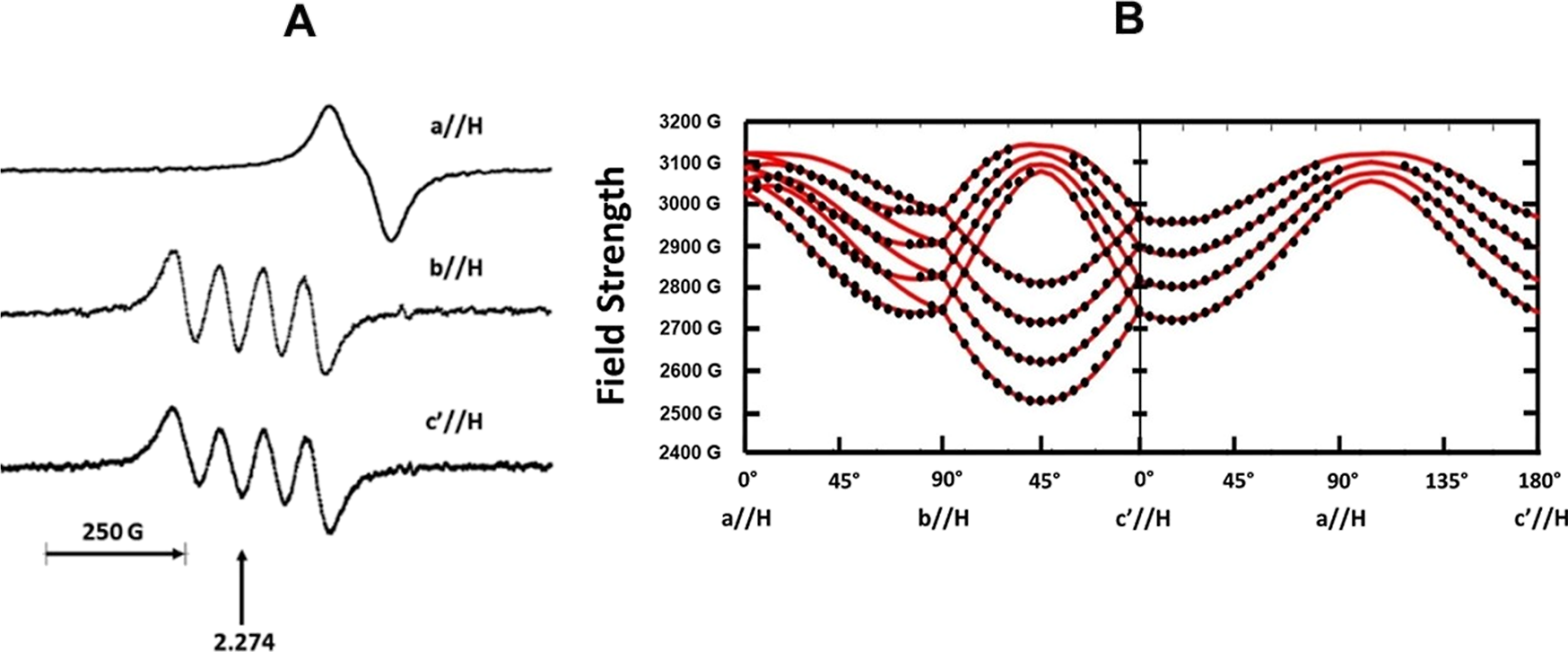
(A) Single crystal X-band EPR spectra of Cu(II)-doped zinc creatininium sulfate hexahydrate (ZnCrnS) acquired at room temperature at the three reference orientations **a**//**H**, **b**//**H** and **c′**//**H**. The widths of individual copper *m*_I_ lines were roughly 30 G. (B) Plot showing the EPR Cu^2+^ pattern resonant roadmap as the crystal in oriented with respect to the external field in the three crystal reference planes. The points are the measured field values. The solid curves represent simulated values using eq 1 and the best-fit **g** and **A**^**C**u^ hyperfine tensors listed in Table 2.

CW-EPR spectra of the doped crystals display a quartet hyperfine pattern characteristic for a Cu^2+^ (*I* = 3/2) coupling. Figure 2A shows the single-crystal EPR spectrum when the external magnetic field **H** is directed along the orthogonal, reference **a**, **b**, and **c′** axes. The copper nuclear split lines are broad and lack any resolved hyperfine splitting. Only one four-line pattern is observed when the external field (**H**) lies in the crystallographic **ac′** plane, which site-splits into two when **H** lies in the **ab** and **bc′** planes due to two symmetry-related sites. The signal pattern orientational symmetry is therefore equivalent to the crystal point group showing consistency with having a single bound copper in the asymmetric unit. The midpoint of the pattern at both **b**//**H** and **c′**//**H** have spectroscopic factor *g* = 2.274 with a resolved copper hyperfine splitting. The nuclear splitting almost entirely collapses at **a**//**H** as the *g*-value decreases. The line widths of the individual single-crystal lines remain constant at 30 G.

The EPR resonant field roadmap is given in Figure 2B as the crystal is rotated in the three reference planes. The assignment of the copper *m*_I_ lines assumed that the maximum hyperfine tensor value was negative. The refined **g** and copper hyperfine (**A**^**Cu**^) tensors that were least-squares fit to the data are listed in Table 2. The rmsd of the data for the fitting was 11.2 MHz. Copper hyperfine values were considered to have similar uncertainty. The solid red curves in the figure show how the theoretical fields varied with crystal orientation using best-fit parameters in eq 1.

**Table 2:** **g** and copper Hyperfine Coupling (**A**^**Cu**^) Tensors Obtained Cu(II)-Doped Zinc Creatininium Sulfate crystals at Room Temperature (295 K)^a^

	Principal Values			Direction Cosines			
				**a**	**b**	**c'**	
**g**	*g*_*z*_	2.446		0.1597	0.6925	0.7064	
	*g*_*y*_	2.112		0.8402	–0.4706	0.2694	
	*g*_*x*_	2.082		0.5182	0.5505	–0.6546	
			(*A*_dipole_)				
**A**^**Cu**^ (MHz)	*A*_*z*_	–327	(−201.7)	0.1686	0.7074	0.6864	
	*A*_*y*_	–59.6	(−65.7)	0.8730	–0.4305	0.2292	
	*A*_*x*_	10.8	(+136.1)	0.4576	0.5606	–0.6902	
		*a*_iso_ = −125.3					
							δ_g_°
Zn-water	Zn-OW3	z		0.2240	0.6740	0.7040	3.7
	Zn-OW1	y		0.8734	–0.4398	0.2089	4.4
	Zn-OW2	x		0.4386	0.6030	–0.6664	5.5
							
							δ_***g***_°
theo. **g**	*g_z_*	*2.278*		*0.1499*	*0.6925*	*0.7057*	*4.3*
	*g_y_*	*2.146*		*0.8854*	*–0.4117*	*0.2159*	*1.9*
	*g_x_*	*2.049*		*0.4401*	*0.5924*	*–0.6748*	*0.7*
							
			(*A*_*dipole*_)				δ_**A**_°
theo. **A**^**Cu**^ (MHz)	*A_z_*	*–495.1*	(−*421*.*5*)	*0.1870*	*0.6944*	*0.6949*	*2.4*
	*A_y_*	*31.2*	(+*104*.*8*)	*0.8875*	*–0.4227*	*0.1836*	*2.0*
	*A_x_*	*243.0*	(+*316*.*6*)	*0.4212*	*0.5824*	*–0.6953*	*2.0*
		*a*_*iso*_ = −*73*.*6*					

powder EasySpin fit		**g**	**A**^**Cu**^ (MHz)
295 K	*g*_*z*_, A_z_	2.443	–312
	*g*_*y*_, A_y_	2.129	–55
	*g*_*x*_, A_x_	2.080	40
			
110 K	*g*_*z,*_ A_z_	2.458	–348
	*g*_*y*_, A_y_	2.109	–29
	*g*_*x*_, A_x_	2.079	52

a The tensor principal directions (direction cosines) refer to the crystallographic **abc′** axes and are compared with the Zn-water directions in the structure. δ_*g*_° and δ_*A*_° are angular deviations between the tensor principal axes and the Zn-water directions. The theoretical values are from DFT calculations (in italic) of the chemical model described in the text using Cu-OW1, Cu-OW2 and Cu-OW3 bond lengths of 2.032, 1.963 and 2.296 Å, respectively. Also listed are the powder **g** and **A**^**Cu**^ principal values determined at 295 and 110 K.

**Figure 3:**
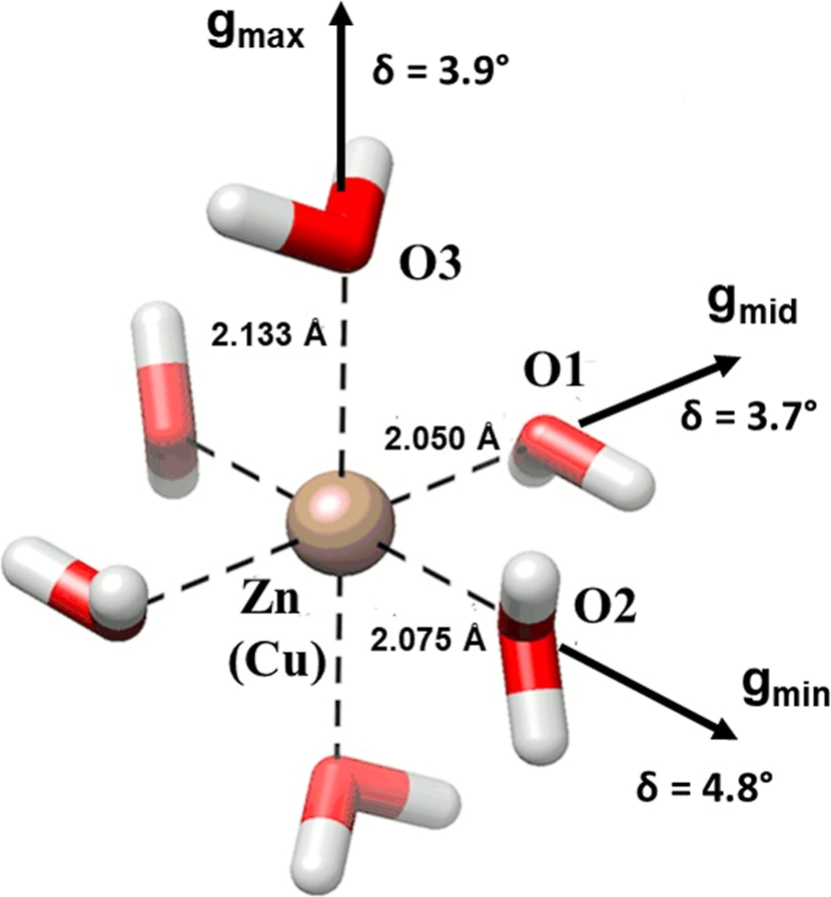
A view of the Zn/Cu hexahydrate complex in the structure of ZnCrnS. Evidence that the copper replaces the zinc in the hexahydrate complex comes from mixed occupancy treatment of the diffraction data. This is confirmed by single crystal EPR measurements which show good agreement between the metal–water directions and the *g*-tensor principal axes. The *g*_min_, *g*_mid_ and *g*_max_ values are associated with the smallest, middle and longest copper–water bonds, respectively. The deviations between these two sets of directions are labeled. The complex was drawn with Chimera.

The **g** and **A**^**Cu**^ tensors are rhombic with principal values: **g**; 2.446, 2.112, 2.082, **A**^**Cu**^; (−327, −59.6, 10.8 MHz) and share the same principal frame having a maximum angular difference of ∼4°. The copper/zinc hexahydrate complex and tensor directions are depicted in Figure 3. A good correlation is found between the tensor axes and the directions of the metal–water bonds in the crystal structure. The deviations (δ_*g*_°: 3.7, 4.4, 5.5°) between the **g** tensor and the metal-OW directions are small and within the uncertainty range for single crystal EPR measurements.^31,79,80^ The tensor values are consistent with those reported in earlier work^31^ as found in previous studies of Cu^2+^(H_2_O)_6_ in doped Tutton salts and analogs,^28−30,81−90^ with a coincidence of *g*_max_ and *A*_max_, and finding that *g*_max_ > *g*_mid_ ≥ *g*_min_ > 2.00, as is expected for a *d*_*x*^2^–*y*^2^_ complex.^91−94^ The lengths of the metal–water bonds in Figure 3 are those of the host ZnCrnS 100 K structure. These change when copper replaces zinc because of Jahn–Teller distortions and other static strains induced by the host lattice, so that, as shown in the pure copper salts, the longest, middle and shortest Cu-OW bond lengths correlate with the *g*_max_ (*g*_*z*_), *g*_mid_ (*g*_*y*_) and *g*_min_ (*g*_*x*_) tensor values, respectively.^79,80,95^ This order is consistent with quantum chemical calculations, as discussed below. The g values are close to the rigid lattice limit^79,80,88,95^ values for Cu^2+^(H_2_O)_6_ found in low temperature EPR studies of Tutton salt systems listed in earlier work (average: *g*_max_ = 2.440, *g*_mid_ = 2.133, *g*_min_ = 2.058),^31^ and pure copper Tutton salts (average: *g*_max_ = 2.430, *g*_mid_ = 2.108, *g*_min_ = 2.062).^79,80,95^ Studies have shown that the principal g values are sensitive to the lengths of the copper–water bonds^93,96,97^ and since the experimental value of the *g* tensor in ZnCrnS are close to the average of the pure copper Tutton salts, the three distances; Cu-OW1, Cu-OW2, Cu-OW3 in doped ZnCrnS were presumed to adopt the average values found in the pure copper Tutton structures (2.032, 1.963, and 2.296 Å),^93^ respectively. As the pure copper creatininium sulfate structure is unknown, the difference between these assumed values and the lengths of the Zn–water bonds in Figure 3 is considered to reflect the extent of local structural adaptation the host lattice undergoes to accommodate copper. Finally, both the g-tensor and copper hyperfine tensor values are very similar to the parameters found in Cd–creatininium sulfate.^31^ However, a major difference in the two crystals is in the alignment of the *g*_min_ direction with the Cu-OW2 bond here, whereas in CdCrnS it is correlated with Cu-OW1.

**Figure 4:**
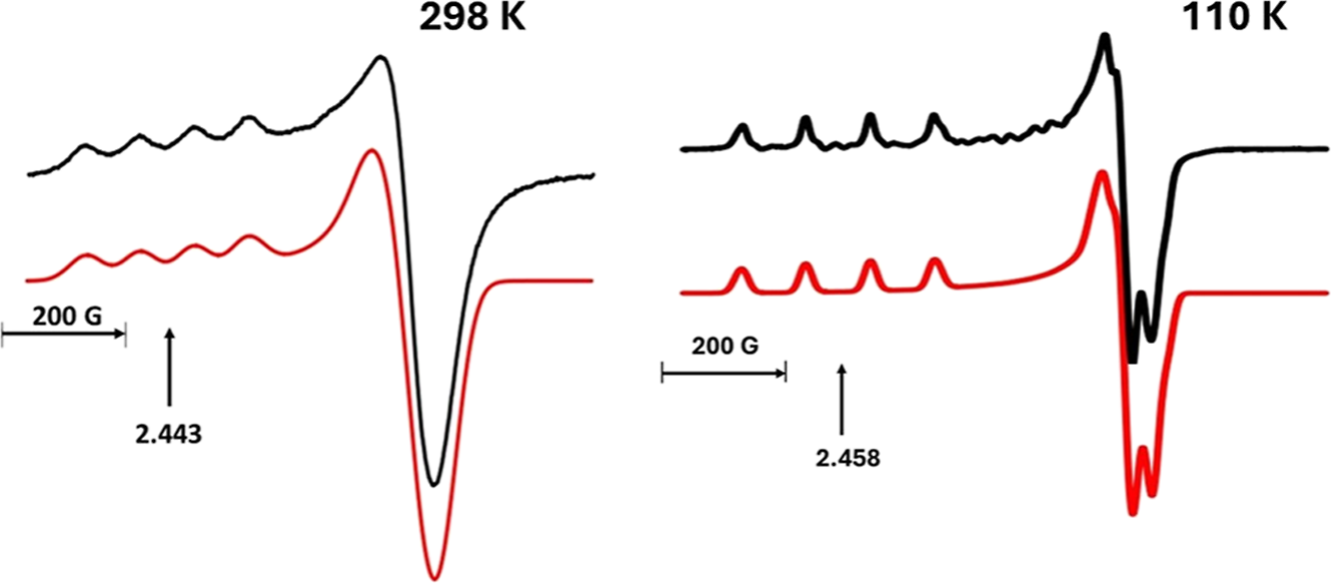
X-band EPR powder spectra of ZnCrnS measured at 298 and 110 K. The red curves are EasySpin simulations of the spectra using the powder parameters contained in Table 2. The g values marked in the figure highlight the slight increase in *g*_max_ of the pattern as the temperature decreases.

The EPR powder spectra observed at 298 and 110 K are shown in Figure 4, matched with the simulations based on EasySpin fits. The spectra show only a slight increase in *g*_max_ (from 2.448 to 2.458), a decrease in *g*_mid_ and a large reduction in line width as the temperature decreases. EasySpin simulations reproduce the spectra using **g**; 2.448, 2.129, 2.080, **A**^**Cu**^; −312, −55.2, 39.8 MHz, Gaussian line width 58 G, for room temperature and **g**; 2.458, 2.109, 2.079, **A**^**Cu**^; −348, −29.2, 52.1 MHz, Gaussian line width 19 G, for 110 K. These are also listed in Table 2. The powder values at room temperature agree with the tensors obtained from single-crystal measurements, that is, within the uncertainty inherent when comparing the spectra of two different sample types.^91^ The small temperature variation of **g** and **A**^**Cu**^ is expected since these are already close to rigid lattice limit values. Similar findings are found in CdCrnS but contrast the significant temperature dependence observed for the copper tensors in all other doped crystals containing copper hexahydrate.^31^

## DFT Calculationsv at the Cu(II) Site

### Electric Field Potential

**Figure 5:**
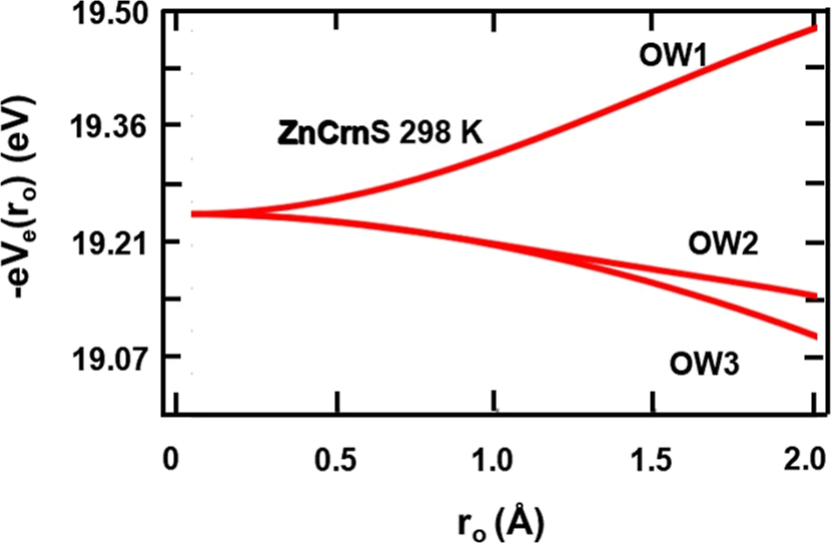
Variation of the potential energy −*eV*_e_(**r**_**o**_) with distance **r**_**o**_ along the three metal–water bonds in doped ZnCrnS for the room temperature structure. Previous results correlate the lower curve line in the plot (OW3) with the longest metal–water bond length and the upper curve (OW1) with the smallest bond length in the copper complex.

Quantum chemical single-point energy calculations using the host lattice atoms were performed using Gaussian 16. These were achieved at the DFT level and used the B3LYP hybrid functional as implemented in the Gaussian package. The chemical model consisted of a cluster of 255 atoms contained in a sphere of about 9 Å centered on the Zn(H_2_O)_6_ complex in the 298 K structure. The basis sets DGDZVP for Zn and 6-311G(d) for H, O, and S were utilized, with a zinc radius of 0.76 Å. The computed Mullikan atomic charges were subsequently used in the electric field potential determinations. The charges in the lattice that generate the internal electric field **E**(**r**) are responsible for the electric potential *V*_e_(**r**), where **E**(**r**) = −grad *V*_e_(**r**). Calculated Mulliken charges were averaged over all the same atoms in the cluster, except for those pertaining to the core host metal–hexahydrate complex. As in a previous approach,^32^ the electrostatic potentials at various points were then determined using a sum of terms; *V*_e_(**r**_**o**_) = *k*_e_∑_*i*_*q*_*i*_/|**r**_**i**_ – **r**_**o**_|, where the *q*_*i*_ are atomic partial charges located at **r**_**i**_ points external to the substituted central Cu^2+^(H_2_O)_6_ complex, and with **r**_**o**_ = 0 specifying the position of copper. The summation included atomic charges contained in a 40 Å sphere centered on the copper. The potential energy of an electron (charge −*e*) placed in this potential is −*eV*_e_(**r**_**o**_). Using this, a map of the electric field potential at distances from the central metal ion along the three metal–water bonds in the complex is shown in Figure 5. The relatively small value for the electric field potential (19 eV 298 K) calculated at the copper site in the ZnCrnS hexahydrate complex is similar to CdCrnS (23 eV 298 K) but is in contrast to the much higher values found in Tutton salt systems (average ∼80 eV).^32^ According to the conclusions from previous work,^32^ this relatively low value predicts a large Silver and Getz model energy gap^28^ of approximately δ_12_ = 600–700 cm^–1^ between copper configurations and therefore a negligible dynamic Jahn–Teller averaging. The finding is consistent with the characteristics of the EPR tensors outlined above. However, the curves in Figure 5 also predict^32,33^ that Cu-OW1 will be the shortest bond length in the complex, Cu-OW2 the intermediate, and Cu-OW3 the longest. This contradicts the order of the relative bond lengths from the experimental g-tensor in Table 2 which shows that Cu-OW2 is the shortest bond and Cu-OW1 is of intermediate length in the complex. The g-tensor determined order is however consistent with the DFT calculations described below.

### Copper Electronic State

The electronic structure of copper was theoretically simulated using ORCA 5.0 employing a chemical model consisting of 67 atoms: the copper-hexahydrate complex, 6 waters and 6 sulfate ions. The geometry of the 6 waters in the hexahydrate was first optimized with Gaussian G16 using DFT at the B3LYP theory level with 6-311G* for all atoms on the Ohio SuperComputer. The optimization gave copper-water (OW1, OW2, OW3) distances of 2.027, 1.985, and 2.187 Å, respectively. These were slightly different from previous theoretical optimizations of copper hexahydrate done in vacuo (2.047, 1.968, 2.296, and 2.05, 1.97, 2.30 Å)^96,97^ as well as the average experimental distances found from the pure copper-tutton salt structures. Therefore, two separate DFT calculations discussed below were performed. One with the current optimized copper-water lengths and the other with the average experimental values. Both calculations gave nearly identical **g** and **A**^**Cu**^ tensors.

**Figure 6:**
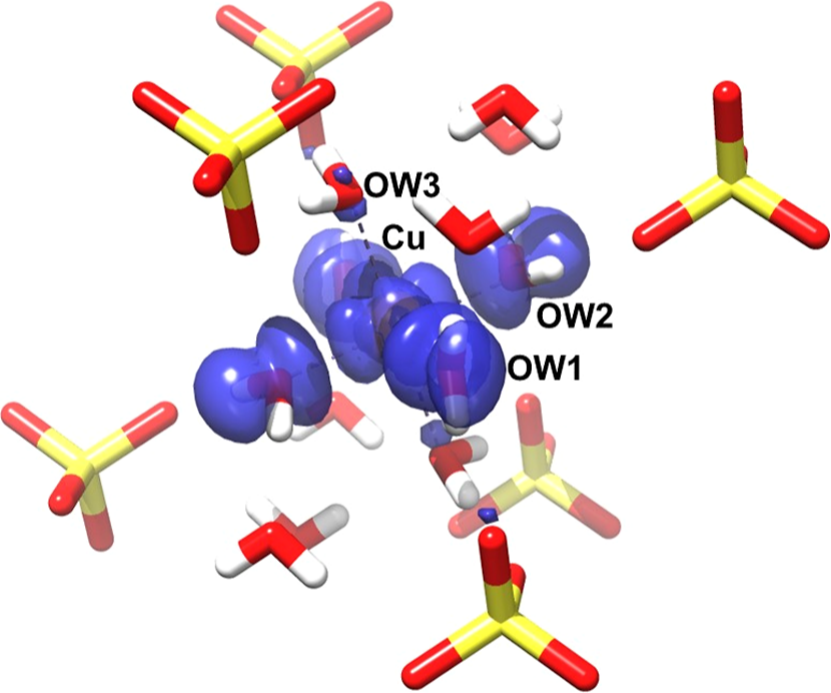
Theoretical spin density distribution over the copper-hexahydrate site in doped ZnCrnS. DFT calculations were performed using the PBE0 functional, IGLOO-III basis for the copper and oxygen, and aug-cc-pVDZ for the rest of the atoms. The model employed Cu-OW1, Cu-OW2 and Cu-OW3 bond lengths of 2.032, 1.963 and 2.296 Å, respectively. The α unpaired spin density surface in blue was rendered with Chimera at a 0.05 contour.

DFT calculations on the electronic state of copper aquo complexes have been evaluated in numerous studies with various functionals.^96,97^ In this work, more recent benchmarking studies^98−101^ guided the choice of functional and atomic basis based on favorable comparison with experimental EPR parameters for a wide range of systems. Consequently, the Perdew–Burke–Ernzerhof (PBE) exchange–correlation hybrid functional PBE0^102,103^ was utilized along with a CP(PPP) basis for the copper, IGLOO-III^104^ for the coordinating atoms (retrieved from basis set exchange^105^), and aug-cc-pVTZ-J for the remaining atoms in the 67 atom model. Calculations with these functional and basis sets were previously found to give good quantitative agreement with experimental EPR spectral parameters in copper complexes.^98,101^ Using these with the experimental Cu-OW bond lengths in the model (Figure 6), generated the **g** and copper hyperfine tensors (**g**: 2.278, 2.146, 2.049, **A**: −495.1, 31.2, 243.0 MHz) listed in Table 2. The tensors show only fair agreement with the measured values but do reproduce their approximate rhombic nature. Notably better is the excellent alignment found between the calculated tensor principal directions and the Cu-OW directions (δ_*g*_: 4.3, 1.9, 0.7°, δ_*A*_: 2.4, 2.0, 2.0°). Overall, the results suggest an unpaired orbital with a large amount of copper *d*_*x*^2^–*y*^2^_ character. In addition, trial calculations that interchange the Cu-OW2 and Cu-OW1 bond lengths led to the expected switch of the theoretical *g*_min_ (*A*_min_) and *g*_mid_ (*A*_mid_) principal directions. The spin density distribution at the copper site derived from the DFT calculations is illustrated in Figure 6.

### Forming the Free Radical

**Figure 7:**
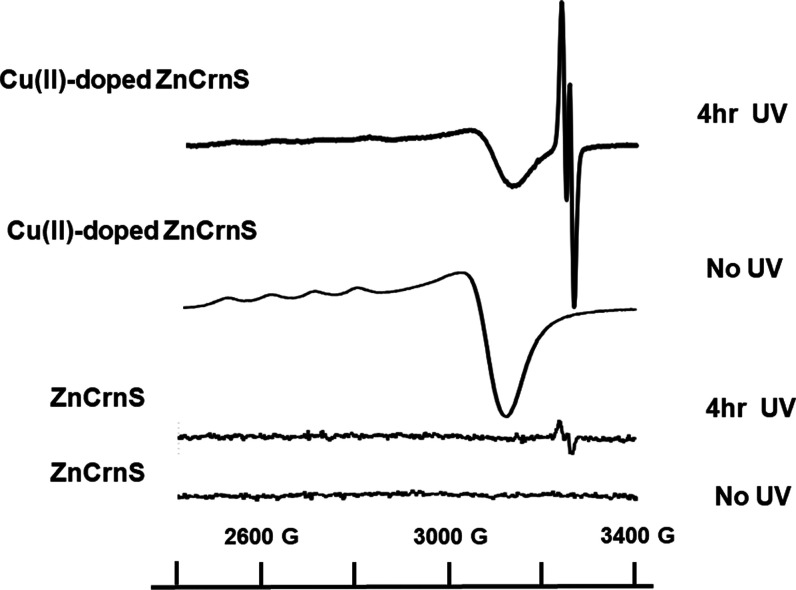
EPR spectra of powdered crystals of ZnCrnS and copper-doped ZnCrnS before and after 4 h UV exposure. The doublet pattern at high field represents the free radical species.

UV-Photoionization of Cu^2+^-doped zinc creatininium sulfate produces a doublet EPR pattern (near the free spin *g*-value 2.002319) at room temperature, signifying a free radical species. Figure 7 shows a comparison of the spectra obtained from the normal and UV-exposed copper-doped vs undoped powdered crystals. No radicals were found in the samples prior to irradiation.

After UV, the free radical EPR signal (peak to peak height) is about 50 times larger in the doped crystalline powders, attesting to the enhancement of UV-sensitization by Cu^2+^. When the UV light is first passed through a U-340 filter, the radical EPR intensity is reduced to ∼3% of the amount without the filter as seen on the right of Figure S1. This reduced intensity matches the 2.7% transmittance at 254 nm measured through the U-340 band-pass filter (Figure S1, left) which demonstrates that 254 nm excitation is exclusively responsible for radical generation.

### Free Radical EPR Analysis

**Figure 8:**
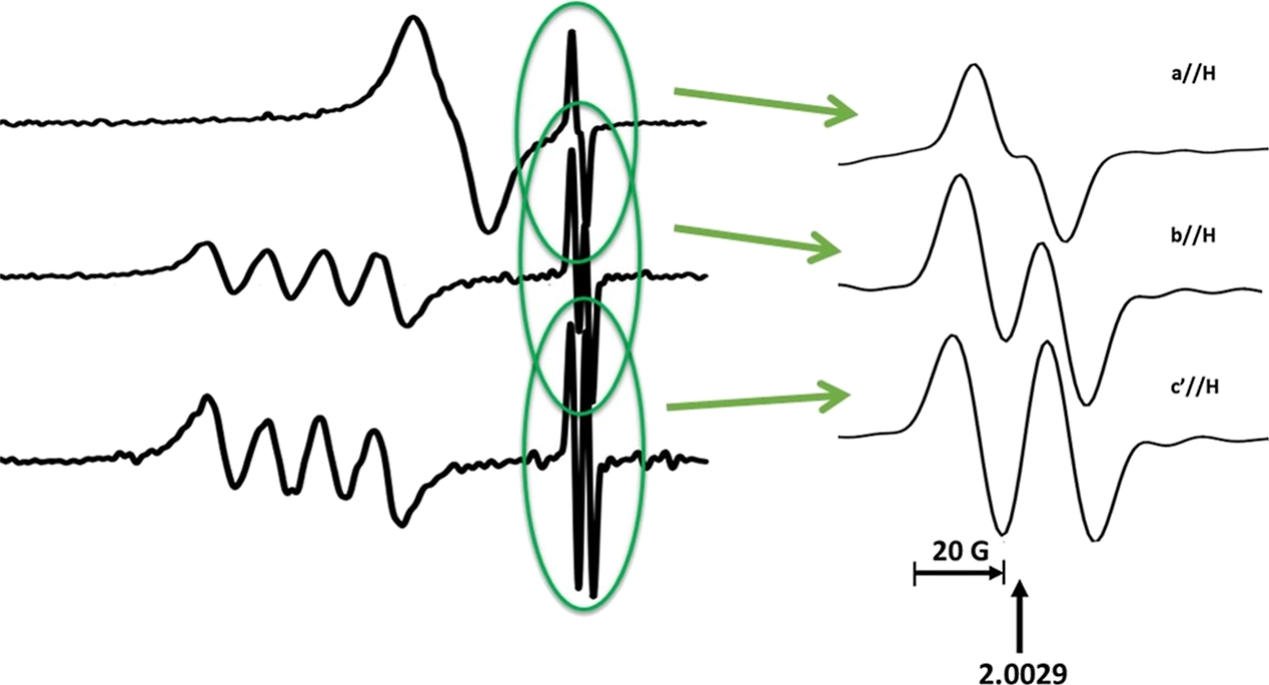
Single crystal X-band EPR spectra of UV-irradiated Cu(II)-doped zinc creatininium sulfate hexahydrate (ZnCrnS) at room temperature at the three reference orientations **a**//**H**, **b**//**H** and **c′**//**H**. The doublet free radical pattern at the three orientations is enlarged on the right.

The EPR single crystal spectra with the external field directed along the three crystal reference orientations is given in Figure 8. The field-expanded view of the free radical doublet pattern is shown to the right in the figure. It is shown below that the doublet splitting arises from a single ^1^H (*I* = 1/2) splitting. There is no evidence for resolved site-splitting of the pattern at any crystal orientation. This is due in part because of unresolved hyperfine couplings as well as a possible contribution of the Cu(II)-dopant electron spin–radical spin interaction to the line width. However, there is a large variation in line broadening. In past EPR studies, similar radicals in X-irradiated creatine and creatininium also showed the absence of measurable site splitting, which limited a detailed orientational analysis of the proton hyperfine tensor.^106^ This difficulty was circumvented in the present work by treating the orientational variation of the broadening as arising from the unresolved site-splitting of a single proton hyperfine coupling. This approach allowed the determination of a full ^1^H coupling tensor.

**Figure 9:**
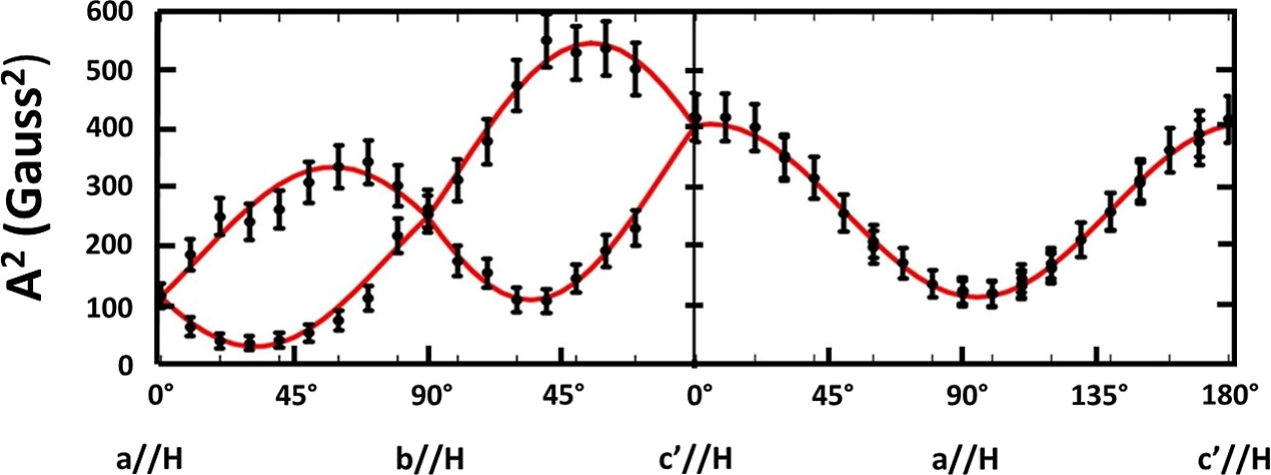
Dependence of the free radical doublet hyperfine splitting-squared (*A*^2^) on the crystal orientation in the external field in the three reference planes. The points are the measured *A*^2^ values using Peakfit and the method described in the text. The solid curves represent simulated values using the fit components of the ^1^H hyperfine tensor listed in Table 2.

In this strategy, Peakfit algorithms were used to first integrate the EPR derivative signal data, and then fit the pattern using either a two- or four-line absorption. In the **ac** plane and along the reference axis, no crystallographic site splitting should exist, and the Peakfit analysis was consistent with a two-line simulation. At crystal orientations that are off-axis, two matched pairs of lines reproduced the pattern. The two pairs reflect the unresolved site splitting. The absorptions that make up each pair were constrained to have the same line width during the fitting procedure. The ^1^H hyperfine splitting at each crystal orientation was then determined as magnetic field differences between the paired lines’ absorption peaks. The square of the measured splittings, denoted *A*^2^ in Figure 9, and their orientational dependencies were employed to determine the ^1^H hyperfine tensor by the Schonland^107^ method, used by Box et al.^108^ Variation in the splitting-squared, *A*^2^, with orientation in the three reference planes was plotted as shown in the figure. Some data comprised average values from independent analyses of repeated EPR experiments which were used to ascertain an estimate of ±1 G uncertainty. Error bars in the plot represent this estimate in the determined splitting. The derived hyperfine values were also assumed to have this uncertainty. The ^1^H hyperfine (**A**^**H**^) tensor that fits the data is listed in Table 3. The solid red curves in the figure show how the tensor in Table 3 fits the squared splitting variation.

The free radical EPR powder spectra observed and simulated at room temperature are pictured in Figure S2. EasySpin was used to fit and simulate the spectrum using **g**; 2.0029, **A**^**H**^; −20.3, −16.5, +7.8 G, with a Gaussian line width of 10.7 G. These are also listed in Table 3. The room-temperature powder values closely agree with the single-crystal measured tensors.

**Table 3:** Free Radical Proton Hyperfine Coupling (**A**) Tensor Obtained from UV-Irradiated Cu(II)-Doped Zinc Creatininium Sulfate Crystals at Room Temperature (295 K) and from Orca DFT Calculations (Italic)^a^

free Radical (g = 2.0029)							
	principal values			direction cosines			
				**a**	**b**	**c'**	
			(*A*_dipole_)				
**A**	*A*_*z*_	–24	(−11) G	0.140	0.596	–0.791	
	*A*_*y*_	–15	(−4) G	0.694	0.510	0.507	
	*A*_*x*_	4.4	(+15) G	–0.706	0.620	0.342	
			a_iso_ = −11.7 G				
							δ_Α_°
radical Geometry	κ	⊥		0.3061	0.4510	–0.8384	13
	η	plane normal		0.5614	0.6257	0.5416	10
	ν	bisector (C–H)		–0.7730	0.6318	0.0577	17
							
			(*A*_*dipole*_)				δ_*Α*_°
Theo. **A**	*A*_*z*_	*–23.3*	(*−8.3*) *G*	*0.2895*	*0.4597*	*–0.8396*	*12*
	*A*_*y*_	*–15.5*	(−*0*.*5*) *G*	*0.5712*	*0.6208*	*0.5369*	*10*
	*A*_*x*_	*–6.3*	(+*8*.*7*) *G*	*–0.7680*	*0.6350*	*0.0829*	*15*
			*a*_*iso*_ = −*15*.*0 G*				

powder EasySpin fit			(*A*_dipole_)
**A**	*A*_*z*_	–20.3	(−10.7) G
	*A*_*y*_	–16.5	(−6.3) G
	*A*_*x*_	7.9	(+14.2) G
			*a*_iso_ = −9.6 G

a The tensor principal directions (direction cosines) refer to the crystallographic **abc′** axes and are compared with the creatininium framework directions in the structure. δ_Α_° are angular deviations between the **A** tensor and these directions. Also listed are the free radical powder **A**^**H**^ principal values determined by EasySpin 295 K.

**Figure 10:**
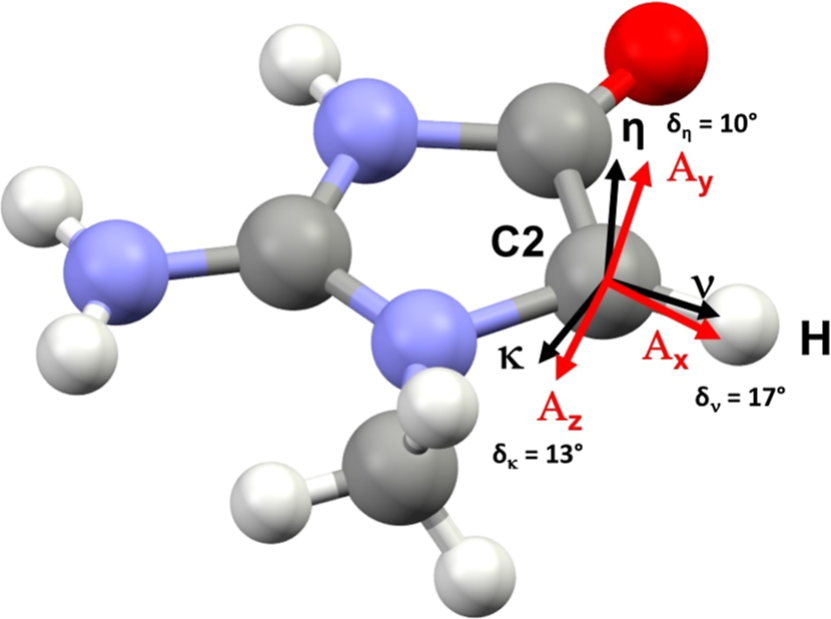
Molecular structure of the creatininium radical in ZnCrnS. The molecular reference directions are *x*: ν along the putative C–H bond, *y*: η normal to the plane N2–C2–C3 and *z*: κ perpendicular to *x* and *y*. The angular deviation between the experimental α-proton hyperfine tensor principal directions and the creatininium frame is indicated by δ.

Correlations between tensor principal axes and molecular structure is a standard tool used to identify free radical species in EPR analysis.^109,110^Figure 10 compares the creatininium structure and the measured principal directions of the proton hyperfine tensor. The molecular reference directions are *x*: ν along the putative C–H bond, *y*: η normal to the plane N2–C2–C3 and *z*: κ perpendicular to *x* and *y*. Under the assumption that the atoms are arranged as in the parent molecule in the unexposed crystal, a good correlation is found between the tensor axes and the creatininium geometry. The deviations (δ_*A*_°: 13, 10, 17°) in directions of the *A*_*x*_, *A*_*y*_, and *A*_*z*_ principal components and the molecular framework are deemed small enough to unambiguously identify the coupling as arising from an α-proton.

α-Proton couplings are well understood from the work of McConnell and his associates^111,112^ on H–C^•^–R π electron radicals. The unpaired electron is situated primarily in a 2p orbital with the axis of symmetry perpendicular to the bonding plane. There is a small negative electron spin density at the adjacent proton ascribed to configuration interaction which, together with a substantial dipole–dipole interaction, accounts for the H_α_ coupling. The isotropic hyperfine component arising from this spin polarization is proportional to the electron spin population on the α-carbon atom as expressed by the McConnell relationship:^111,112^*a*_iso_ = **Q**ρ_c_, where *a*_iso_ is the isotropic hyperfine coupling, ρ_c_ is the electron density at the central (α) carbon, and **Q**, is a constant with a value of approximately −23.0 G MHz, the exact value depending on the environment of the unpaired electron. Using *a*_iso_ from Table 3 gives a spin density of 0.5 on C2. Simple analytical expressions for the carbon orbital and hyperfine dipolar interaction predict that the maximum positive dipolar hyperfine coupling (*A*_*x*_) is along the C–H_α_ bond direction, and the intermediate dipolar coupling (*A*_*y*_) is normal to the bonding plane.^112,113^ More recent theoretical DFT calculations have shown that the maximum dipolar direction is a reliable indicator of the C–H_α_ bond direction, even in nonplanar radical systems.^114^ As the figure illustrates, the ^1^H coupling tensor reported in Table 3 is in accordance with this model.

### Free Radical DFT Characterization

**Figure 11:**
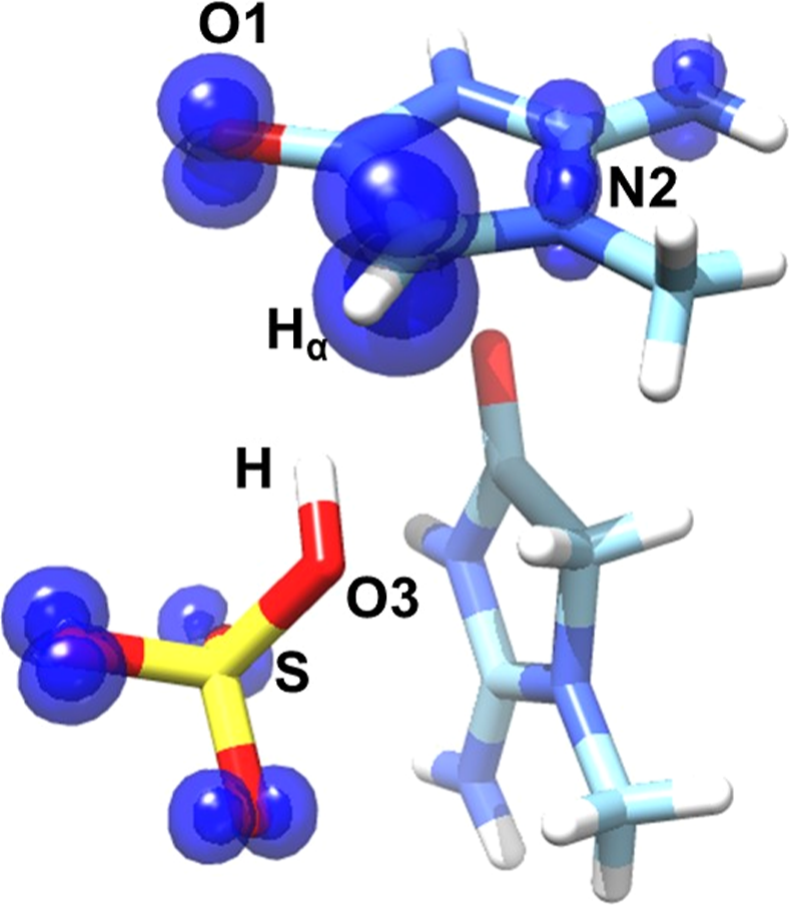
Theoretical spin density distribution of the free radical in UV exposed Cu-doped ZnCrnS. Orca 5.0 was used at the DFT level using the B3LYP functional with 6-311++G(p,d) basis sets for all atoms. The model employed optimized bond lengths; C2–H_α_ = 1.07 Å and S–O3 = 1.66 Å and O3–H = 0.99 Å. The spin density isosurface in blue was rendered with Chimera at a 0.04 contour.

DFT calculations on the free radical were carried out with ORCA using the B3LYP functional with 6-311++G(p,d) basis sets for all atoms. The chemical model is shown in Figure 11 and consists of a creatinine cation radical, a bisulfite ion and an additional creatininium ion. Calculations assume that the N2–C2–C3 atoms of the radical remain in the same plane as in the undamaged molecule and that the C–H_α_ bond direction bisects the N2–C2–C3 bond angle. Furthermore, this model proposes that the C2–H2A proton moves to hydrogen-bonded sulfate O3 when the radical forms. The length of the radical C2–H_α_ bond and position of the hydrogen in the formed sulfite were first determined by geometry optimization using Gaussian G16 at the DFT level with the B3LYP functional initially for a 75 atom model using the 6-311++G** basis for Zn, S and 6-311G for C O N H, and then later with a 134 atom model using the G-31G* basis for additional atoms in the structure to include possible hydrogen bond acceptors for the sulfite hydrogen. The optimizations gave a C2–H length of 1.07 Å and placed the sulfite O3-hydrogen (S–O3 length = 1.66 Å, O3–H 0.99 Å, S–O3–H angle = 112°) directed toward the creatininium radical O1, making a weak interaction (O3–H···O1 distance = 3.42 Å). Using these parameters in the model shown below, the H_α_ hyperfine tensor was calculated and is included in Table 3. The resulting spin density distribution (isosurface at the 0.04 contour level) is shown in Figure 11. The theoretical H_α_ hyperfine tensor (−23.3, −15.5, −6.3 G) shows good agreement with both experimental values (−24, −15, +4.4 G) and principal directions (δ_*A*_: 12, 10, 15°).

### Correlation between the Free Radical and Cu^2+^ EPR Signals

**Figure 12:**
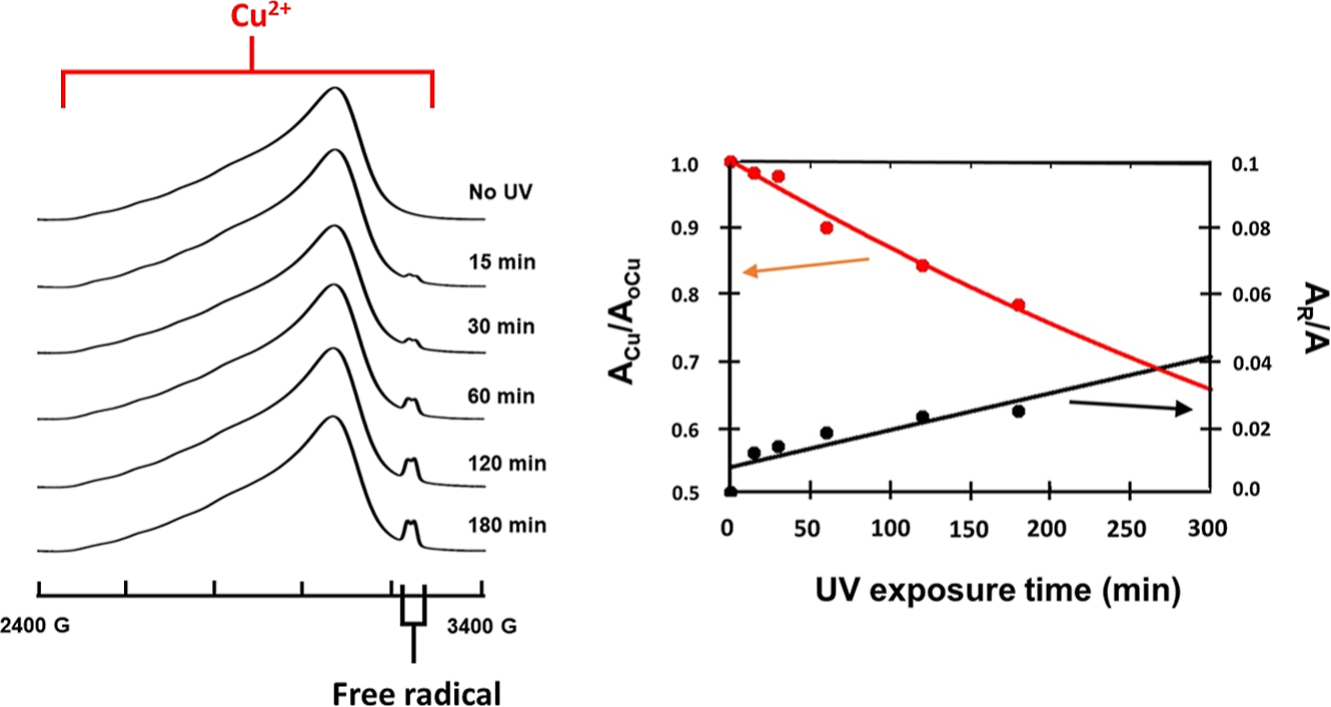
Correspondence between the growth in the creatininium free radical and the decay of the Cu(II) species upon UV exposure. (Left) Integrated EPR spectra of powdered crystalline doped ZnCrnS at various exposure times. (Right) Plot of the relative amounts of Cu(II) and free radical species vs UV exposure time, where **A**_**Cu**_ = area of the copper EPR pattern, **A**_**o**_ = area of the original copper EPR pattern, **A**_**R**_ = area of the free radical pattern and **A** = area of the entire EPR pattern.

There is a correspondence between the growth in the creatininium free radical concentration and the decay of the Cu(II) pattern upon UV exposure. Figure 12 shows the integrated EPR patterns of the copper-doped exposure, Cu(II) is reduced to 65% of its original concentration and the free radical will have grown to approximately 4% of the total EPR pattern, or about six Cu(II) are reduced for every radical generated.

## Discussion

The measured g-tensors of the copper hexahydrate complex in the doped Cd and Zn creatininium sulfate crystals are almost identical. Their major difference being a switch in their *g*_min_ and *g*_mid_ associated directions, that is, in their presumptive relative copper–water bond lengths. The fact that *g*_min_ is correlated with the smallest Cu-OW bond length has been borne out in numerous previous studies and is verified by current DFT calculations. Although the host crystal metal-water distances are very different in the two, the doped copper-water distances must be closely similar, attesting to the dominant static Cu(II) and lattice distortions in the hexahydrate complexes. Our previous results using the electric field gradient fail to predict the order of the relative copper–water bond lengths, although this is a successful prognosticator for all of the doped Tutton salts studied as well as for CdCrnS. A possible reason for this omission could be a local structural change in the position of the sulfate about the copper-hexahydrate complex that, if present, does not significantly alter the trend found in these other systems. Previous work has shown that the first shell ions make a major contribution to the electric field gradient at the complex. Any slight change in their position and/or partial charges could have a large impact on the relative field gradient along the three water bonds^32,33^ which could possibly lead to a switch in the OW1 and OW2 curves in Figure 5. The temperature insensitivity of the EPR spectra along with near rigid-lattice limit tensor values at room temperature shows that the Cu(II) hexahydrate in the Zn structure, like in CdCrnS, has little dynamic character. This is consistent with our earlier analysis^32^ which showed that little or no dynamic effects will manifest when a small electric field gradient exists at the Cu(II) position compared to values found in the Tutton salt systems.

A plausible reason for the lack of the Cu(II) EPR temperature dependence in ZnCrnS compared to the Tutton salts is the marked inequivalence of the water environments for the three metal bound waters of the Zn(H_2_O)_6_ complex, since they are involved in different intermolecular interactions. This is described in most works as host lattice forces or strains.^28,30^ Accordingly, these local strain interactions could be different enough in Cu^2+^-doped ZnCrnS compared to the Tutton salt systems to significantly reduce any temperature dependence of the copper EPR parameters. One major difference is the change of the water–monocation interaction for the longest and intermediate length metal-OW waters in the Tutton salt structures with a water–water hydrogen bond for all three metal bound waters in ZnCrnS. These geometric distortions and lattice fields at the Cu(H_2_O)_6_ octahedral complex lift the degeneracy of the *e*_g_-electronic states. If the splittings due to this lowering of the complex symmetry exceed the energy of Jahn–Teller interactions, the Jahn–Teller effect, and any associated dynamics, will be depressed.^33,81^

The electronic state of Cu(II) in ZnCrnS has been theoretically determined by DFT calculations. A Mulliken analysis gives an 84% spin population on the copper and 3–5% each on the in-plane oxygen ligands, residing primarily in the equatorial orbitals of the complex. This is consistent with the experimental EPR parameters which give evidence for a predominantly *d*_*x*^2^–*y*^2^_ unpaired orbital^92^ and agrees with the spin density distribution profile illustrated in Figure 6.

A creatininium free radical is generated in doped ZnCrnS by 254 nm illumination. The ^1^H hyperfine tensor measured by EPR has the characteristics for an alpha-proton coupling with a spin density less than one on the C_α_ atom.^109,110,114^ DFT calculations of a creatinine cation radical give EPR characteristics that match those experimentally determined, i.e., the theoretical unpaired spin residing mostly (62%. Mulliken) on the C2 carbon, with the remaining delocalized over the creatininium molecule; ∼20% on O1, and less on N2 and C1, and a residual amount on the three distant sulfite oxygens.

This species is similar to the radicals observed at room temperature in X-irradiated creatine^106,115^ and creatinine crystals.^106^ The derived unpaired spin distribution in these is consistent with the creatininium radical characteristics found here. In the presence of doped Cu(II) in the crystal, the free radical yield is enhanced by about 50 times, suggesting that its reduction is necessary for the stabilization of the creatininium radical. However, there is no 1:1 quantitative relationship since the EPR spectral integration shows that multiple Cu(II)’s are reduced for every radical formed. There is also no evidence of the presence of any other radical species at room temperature after UV irradiation. It is possible that other oxidation products are initially created but lead to diamagnetic products via secondary processes.^109^ Previous EPR studies on UV-exposed Cu(II)-doped amino acid crystals also found a decrease in the copper signal upon free radical formation. These studies proposed^34−36^ that cationic radicals are formed on the copper-ligated molecules by a ligand-to-metal charge transfer (LMCT) process.^116^

In contrast, the mechanism of radical formation and copper reduction in the present system is interpreted using a photoexcited proton-coupled electron transfer (PCET)^117^ model. An alternative scheme of a direct homolytic cleavage of a C2 hydrogen by the 254 nm light (4.88 eV) to form the stable radical appears less likely given its negligible yield in the Cu^2+^ absent crystals.

**Figure 13:**
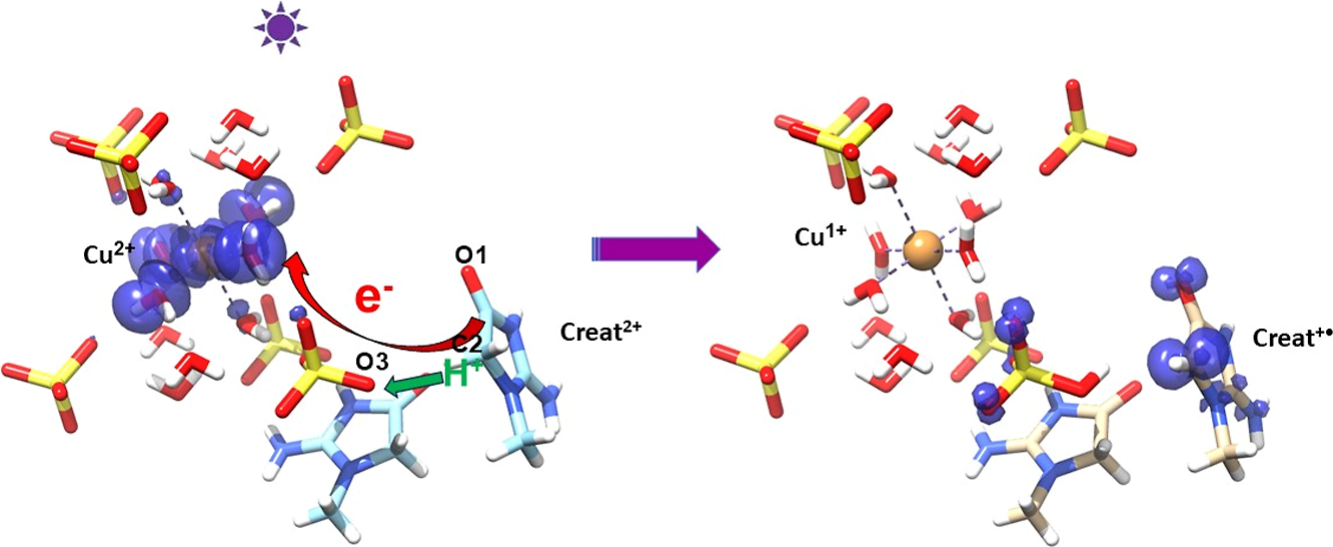
Proposed structural scheme for the excited-state PCET mechanism involves a creatininium electron being promoted by the 254 nm light that enables it to tunnel through the C2–H2A···O3–S interaction to the sulfate and from there through a hydrogen bonded water of the hexahydrate complex to copper. Cu(II) is thus reduced to Cu(I) while the creatinine cation radical is stabilized by transferring its H2A proton to O3 forming a sulfite. The nonbonded distance between C2 and Cu(II) for the site shown is 8.37 Å.

The crystal structure offers a plausible pathway for PCET and is illustrated in Figure 13. Upon excitation, a creatininium electron is transferred to copper possibly by tunneling through the C–H···O–S interaction to the sulfate and then through hydrogen-bonded water from the hexahydrate complex. This would reduce Cu(II) to Cu(I) and, while transiently forming an inherently unstable creatinine dication radical, is then complemented by the release of the H2A proton. This release is facilitated by transferring the proton to the oxygen of the sulfate involved in the C2–H2A···O3 hydrogen bond. A slight turn of the sulfite hydrogen to cause a weak interaction with the parent O1 serves to further stabilize the radical and hinder a back reaction. The DFT results indicate a spread of the spin density of the creatininium radical onto the remote oxygens of the newly formed sulfite that are hydrogen bonded to the hexahydrate. This delocalization might partly reflect the pathway for the promoted electron transfer to copper. This scheme is quite similar to the photoexcited direct electron transfer and deprotonation between a tryptophan and the copper(II) center proposed in UV-exposed Azurin mutants.^118^ There, the promoted electron from the indole π-system of Trp-48 is suggested to tunnel through the backbone to the copper center by either or both of two pathways, both of which require the electron to also tunnel through hydrogen bonds to reach the type I copper site. The long-lived tryptophan radical is stabilized by deprotonation of the cation radical.

In Azurin, the nonbonded distance from the W48 indole to copper ∼10 Å. In ZnCrnS, there are three different metal hexahydrate complexes near the creatininium that communicate via hydrogen bonds with the sulfite ion. These are depicted in Figure S3. A copper ion replaced in any of these provides a potential reduction site. The metal-to-creatinine C2 distances are all slightly different; 8.32, 8.37 and 9.76 Å, but are shorter than the tryptophan-to-Cu^2+^ distance in Azurin.

## Conclusions

One of the objectives of this investigation is to determine the crystal structure of another metal organic hybrid analog of Tutton salt and to gain a greater understanding of the relationship between dynamics and the electronic nature of copper hexahydrate.

X-ray diffraction experiments determined the crystal structure of ZnCrnS at both 100 and 298 K. ZnCrnS, like all Tutton salts, crystallizes in the monoclinic space group *P*2_1_/*n*. The unit cell contains two zinc hexahydrate complexes, four creatininium ions, four sulfates and four additional solvation waters. The structure is isomorphous to the Cd/Cu creatininium sulfate.

Single crystal EPR measurements at room temperature of doped crystals determined the **g** and copper hyperfine (**A**^**Cu**^) tensors (principal values: **g**; 2.446, 2.112, 2.082, **A**^**Cu**^; −327, −59.5, 10.8 MHz). The EPR spectra of the powder at room temperature gave **g**; 2.443, 2.129, 2.080 and **A**^**Cu**^; −312, −55, 40 MHz, and at 110 K gave **g**; 2.458, 2.109, 2.079 and **A**^**Cu**^; −348, −29, 52 MHz. The room temperature tensors are close to the rigid-lattice limit values found in copper-doped Tutton salts and display only minimal changes at low temperature, indicating negligible copper dynamics. This finding is consistent with the low lattice electric field gradient value calculated at the metal site, which has been proposed as a measure of dynamic effects in copper hexahydrate systems. However, in the copper-doped metal creatinine sulfate crystals, a possible explanation for this absence of dynamics could be the different local lattice strain interactions involving the metal water environments as compared to the Tutton salt systems. Both crystallography and EPR results find doped copper replacing zinc in the structure. A significant difference found between the copper-doped Zn and Cd creatinine systems is a switch in the Cu-OW bond corresponding to the minimum g-tensor direction, reflecting a difference in their local structural adjustment when copper dopes in the two systems.

EPR spectroscopy shows the formation of a free radical doublet pattern when copper-containing samples are exposed to 254 nm UV light. The number of free radicals formed increases somewhat linearly with irradiation time and is correlated with a decrease in the amplitude of the copper EPR signal. The radical species is identified as carbon-centered on the creatinine moiety, which is created by the oxidative release of one of its methylene hydrogens. A pathway can be postulated for the creation of the radical as part of an excited-state proton-coupled electron transfer from the creatininium cation to Cu(II). This scheme involves a concomitant reduction of Cu(II) to Cu(I) upon UV-induced oxidation of the creatininium ion, where the radical and electron transfer are stabilized by a methylene-proton transfer to a neighboring sulfate oxygen. The theoretical background and importance of PCET in chemical processes,^119−121^ and more specifically in hole/electron transfer in biological molecules by radical formation,^122^ has been nicely reviewed. The enhancement of photoexcited PCET processes by nearby Cu^2+^ ions that result in radical species, such as observed here, may have important considerations both in biological systems and in the design of advanced functional materials.

## Supporting Information Available

Tables S1–S6 (crystallographic data) for the 298 K structure and Tables S7–S12 (crystallographic data) for the 100 K structure. Figure S1 (left) shows the Biomate-3 transmittance measurement of the U-340 filter superimposed on the emission spectra of the Pen-ray Hg lamp and (right) the room temperature EPR spectra of UV exposed powders of ZnCrnS with and without passage through the U-340. Figure S2 is the experimental and EasySpin simulated powder EPR spectra of the free radical pattern. Figure S3 is a view of the structure of the 3 metal sites hydrogen bonded to the sulfate ion that is postulated to accept the C2H proton when the creatininium radical forms (PDF)
298 K (rt_a.cif) structure (CIF)
100 K (100k_a.cif) structure (CIF)

## Supplementary Material

jp4c06133_si_001.pdf

jp4c06133_si_002.cif

jp4c06133_si_003.cif
