# Supplementary material for: Cu(II) Stability and UV-Induced Electron Transfer in a Metal–Organic Hybrid: An EPR, DFT, and Crystallographic Characterization of Copper-Doped Zinc Creatininium Sulfate
Source: J Phys Chem A. 2024 Nov 20;128(48):10380–94. doi: 10.1021/acs.jpca.4c06133 (PMC11626505; doi:10.1021/acs.jpca.4c06133)
Supplement: Supplementary file 1 — jp4c06133_si_001.pdf [file jp4c06133_si_001.pdf]

Supporting Information

Cu(II) stability and UV-Induced Electron Transfer in a Metal-Organic Hybrid: An EPR, DFT and Crystallographic Investigation of Copper-Doped Zinc Creatininium Sulfate

Michael J. Colaneri[†*], Simon J. Teat[§] and Jacqueline Vitali[‡*]

Department of Chemistry and Physics, State University of New York at Old Westbury, Old Westbury, New York 11568, Lawrence Berkeley National Lab, 1 Cyclotron Road MS 15RO317, Berkeley, CA 94720, Department of Physics and Department of Biological, Geological and Environmental Sciences, Cleveland State University, Cleveland, Ohio 44115

*Authors to whom correspondence should be addressed. E-mail: colanerim@oldwestbury.edu Mailing Address: Department of Chemistry and Physics, SUNY at Old Westbury, Old Westbury, NY 11568, USA, Tel: (516) 876-2756. E-mail: j.vitali@csuohio.edu Mailing Address: Department of Physics and Department of Biological, Geological and Environmental Sciences, Cleveland State University, Cleveland, OH 44115, USA, Tel: (216) 687-2431.

†SUNY at Old Westbury.

§Lawrence Berkeley National Lab.

‡Cleveland State University.

Tables for 298K structure

Table S1. Crystal data and structure refinement for the 298K structure (JV_ZnCu_25C_a).

Identification code	JV_ZnCu_25C_a	
Empirical formula	C8 H32 Cu0.04 N6 O18 S2 Zn0.96	
Formula weight	629.81	
Temperature	298(2) K	
Wavelength	0.7288 Å	
Crystal system	Monoclinic	
Space group	$P2_1/n$	
Unit cell dimensions	a = 6.4433(15) Å	α= 90°.
	b = 28.051(7) Å	β= 110.934(9)°.
	c = 7.1452(17) Å	γ = 90°.
Volume	1206.2(5) $Å^3$	
Z	2	
Density (calculated)	1.734 Mg/m^3	
Absorption coefficient	1.365 mm^{-1}	
F(000)	656	
Crystal size	0.300 x 0.250 x 0.200 mm^3	
Theta range for data collection	2.979 to 30.016°.	
Index ranges	-8<=h<=8, -38<=k<=38, -9<=l<=9	
Reflections collected	40031	
Independent reflections	3264 [R(int) = 0.0604]	
Completeness to theta = 25.930∞	99.9 %	
Absorption correction	Semi-empirical from equivalents	
Max. and min. transmission	0.772 and 0.686	
Refinement method	Full-matrix least-squares on F^2	
Data / restraints / parameters	3264 / 0 / 218	
Goodness-of-fit on F^2	1.116	
Final R indices [I>2sigma(I)]	R1 = 0.0316, wR2 = 0.0771	
R indices (all data)	R1 = 0.0354, wR2 = 0.0803	
Extinction coefficient	0.039(2)	
Largest diff. peak and hole	0.623 and -0.551 e.$Å^{-3}$	

Table S2. Fractional atomic coordinates (x 10^4) and equivalent isotropic displacement parameters ($Å^2$x 10^3) for JV_ZnCu_25C_a. U(eq) is defined as one third of the trace of the orthogonalized U^{ij} tensor.

	x	y	z	U(eq)
Zn1	10000	5000	0	20(1)
Cu1	10000	5000	0	20(1)
O1W	6960(2)	5319(1)	-610(2)	33(1)
O2W	9456(2)	4555(1)	2107(2)	30(1)
O3W	11651(2)	5519(1)	2253(2)	25(1)
S1	6884(1)	6154(1)	3313(1)	22(1)
O2	5287(2)	5896(1)	1553(2)	29(1)
O3	6118(2)	6644(1)	3330(2)	35(1)
O4	6874(2)	5900(1)	5138(2)	36(1)
O5	9095(2)	6146(1)	3196(2)	44(1)
N1	3018(2)	6870(1)	4953(2)	26(1)
C1	3037(2)	6667(1)	6687(2)	24(1)
N2	1604(2)	6879(1)	7356(2)	29(1)
C2	490(3)	7266(1)	6003(3)	32(1)
C3	1542(3)	7243(1)	4429(2)	32(1)
O1	1215(3)	7502(1)	2997(2)	52(1)
N3	4336(3)	6306(1)	7521(2)	34(1)
C4	1005(3)	6728(1)	9042(3)	42(1)
O4W	3316(2)	5091(1)	-3921(2)	29(1)

Table S3. Bond lengths [Å] and angles [°] for JV_ZnCu_25C_a.

Zn1-O1W#1	2.0531(12)
Zn1-O1W	2.0532(12)
Zn1-O2W	2.0790(12)
Zn1-O2W#1	2.0790(12)
Zn1-O3W#1	2.1480(11)
Zn1-O3W	2.1481(11)
O1W-H1WA	0.77(3)
O1W-H1WB	0.79(2)
O2W-H2WA	0.84(3)
O2W-H2WB	0.75(3)
O3W-H3WA	0.83(2)
O3W-H3WB	0.85(3)
S1-O5	1.4563(12)
S1-O3	1.4626(12)
S1-O4	1.4877(13)
S1-O2	1.4951(11)
N1-C1	1.3588(19)
N1-C3	1.373(2)
N1-H1N	0.90(2)
C1-N3	1.314(2)
C1-N2	1.3228(19)
N2-C4	1.454(2)
N2-C2	1.460(2)
C2-C3	1.508(2)
C2-H2A	0.99(2)
C2-H2B	0.96(2)
C3-O1	1.211(2)
N3-H3NA	0.77(2)
N3-H3NB	0.85(2)
C4-H4A	0.9600
C4-H4B	0.9600
C4-H4C	0.9600
O4W-H4WA	0.79(3)
O4W-H4WB	0.73(3)

O1W#1-Zn1-O1W	180.0
O1W#1-Zn1-O2W	88.85(5)
O1W-Zn1-O2W	91.15(5)
O1W#1-Zn1-O2W#1	91.15(5)
O1W-Zn1-O2W#1	88.85(5)
O2W-Zn1-O2W#1	180.00(7)
O1W#1-Zn1-O3W#1	92.51(5)
O1W-Zn1-O3W#1	87.49(5)
O2W-Zn1-O3W#1	88.27(5)
O2W#1-Zn1-O3W#1	91.73(5)
O1W#1-Zn1-O3W	87.49(5)
O1W-Zn1-O3W	92.51(5)
O2W-Zn1-O3W	91.73(5)
O2W#1-Zn1-O3W	88.27(5)
O3W#1-Zn1-O3W	180.0
Zn1-O1W-H1WA	127.8(18)
Zn1-O1W-H1WB	121.5(16)
H1WA-O1W-H1WB	109(2)
Zn1-O2W-H2WA	119.9(17)
Zn1-O2W-H2WB	115(2)
H2WA-O2W-H2WB	110(3)
Zn1-O3W-H3WA	116.2(16)
Zn1-O3W-H3WB	114.3(16)
H3WA-O3W-H3WB	105(2)
O5-S1-O3	110.77(8)
O5-S1-O4	110.71(8)
O3-S1-O4	109.54(7)
O5-S1-O2	109.76(7)
O3-S1-O2	109.13(7)
O4-S1-O2	106.83(7)
C1-N1-C3	110.29(13)
C1-N1-H1N	122.0(14)
C3-N1-H1N	127.4(14)
N3-C1-N2	126.61(15)
N3-C1-N1	122.20(14)

N2-C1-N1	111.19(13)
C1-N2-C4	126.46(14)
C1-N2-C2	109.67(13)
C4-N2-C2	123.55(14)
N2-C2-C3	102.43(12)
N2-C2-H2A	112.2(13)
C3-C2-H2A	110.9(13)
N2-C2-H2B	109.1(13)
C3-C2-H2B	112.4(13)
H2A-C2-H2B	109.7(18)
O1-C3-N1	125.65(17)
O1-C3-C2	127.94(16)
N1-C3-C2	106.39(13)
C1-N3-H3NA	119.4(16)
C1-N3-H3NB	118.6(16)
H3NA-N3-H3NB	122(2)
N2-C4-H4A	109.5
N2-C4-H4B	109.5
H4A-C4-H4B	109.5
N2-C4-H4C	109.5
H4A-C4-H4C	109.5
H4B-C4-H4C	109.5
H4WA-O4W-H4WB	104(3)

Symmetry transformations used to generate equivalent atoms:

#1 -x+2,-y+1,-z

Table S4. Anisotropic displacement parameters ($Å^2$x 10^3) for JV_ZnCu_25C_a. The anisotropic displacement factor exponent takes the form: $-2\pi^2[h^2 a^{*2}U^{11} + ... + 2 h k a^* b^* U^{12}]$

	U^{11}	U^{22}	U^{33}	U^{23}	U^{13}	U^{12}
Zn1	18(1)	25(1)	18(1)	0(1)	6(1)	2(1)
Cu1	18(1)	25(1)	18(1)	0(1)	6(1)	2(1)
O1W	22(1)	45(1)	27(1)	-11(1)	4(1)	10(1)
O2W	29(1)	36(1)	26(1)	7(1)	13(1)	4(1)
O3W	24(1)	27(1)	25(1)	-3(1)	11(1)	-1(1)
S1	20(1)	21(1)	24(1)	-3(1)	9(1)	2(1)
O2	28(1)	34(1)	24(1)	-6(1)	9(1)	-3(1)
O3	42(1)	22(1)	48(1)	-2(1)	25(1)	5(1)
O4	43(1)	38(1)	24(1)	6(1)	10(1)	10(1)
O5	25(1)	47(1)	66(1)	-19(1)	23(1)	-4(1)
N1	28(1)	25(1)	24(1)	0(1)	10(1)	2(1)
C1	25(1)	23(1)	24(1)	-2(1)	8(1)	0(1)
N2	30(1)	29(1)	30(1)	0(1)	14(1)	6(1)
C2	30(1)	29(1)	35(1)	-1(1)	9(1)	8(1)
C3	34(1)	28(1)	30(1)	1(1)	8(1)	4(1)
O1	66(1)	49(1)	43(1)	21(1)	21(1)	22(1)
N3	43(1)	32(1)	33(1)	9(1)	20(1)	15(1)
C4	49(1)	47(1)	37(1)	2(1)	26(1)	6(1)
O4W	32(1)	32(1)	23(1)	2(1)	9(1)	3(1)

Table S5. Fractional hydrogen coordinates (x 10^4) and isotropic displacement parameters ($Å^2$x 10^3) for JV_ZnCu_25C_a.

	x	y	z	U(eq)
H1WA	6560(40)	5464(8)	110(40)	45(6)
H1WB	5940(40)	5256(8)	-1580(40)	39(6)
H2WA	10520(50)	4409(9)	2930(40)	50(7)
H2WB	8740(50)	4659(9)	2630(40)	55(8)
H3WA	10840(40)	5721(8)	2500(30)	40(6)
H3WB	12640(40)	5680(9)	2000(40)	51(7)
H1N	3960(40)	6778(8)	4350(30)	42(6)
H2A	760(40)	7579(8)	6680(30)	44(6)
H2B	-1080(40)	7203(8)	5460(30)	39(5)
H3NA	4350(40)	6208(8)	8540(30)	35(6)
H3NB	5140(40)	6187(8)	6920(40)	46(6)
H4A	456	6996	9560	109(12)
H4B	2291	6599	10073	95(10)
H4C	-128	6487	8607	127(14)
H4WA	3350(40)	4819(10)	-4190(40)	47(7)
H4WB	3010(40)	5212(9)	-4890(40)	48(7)

Table S6. Hydrogen bonds for JV_ZnCu_25C_a [Å and °]. The criteria used are those of the program PLATON[1,2].

D-H...A	d(D-H)	d(H...A)	d(D...A)	<(DHA)
O1W-H1WA...O2	0.77(3)	1.95(3)	2.7147(17)	171(2)
O1W-H1WB...O4W	0.79(2)	1.96(2)	2.7473(18)	177(2)
O2W-H2WA...O4#2	0.84(3)	1.95(3)	2.7878(18)	177(2)
O2W-H2WB...O4W#3	0.75(3)	1.99(3)	2.7399(18)	177(3)
O3W-H3WA...O5	0.83(2)	1.83(2)	2.6567(17)	176(2)
O3W-H3WB...O2#4	0.85(3)	1.94(3)	2.7745(17)	166(2)
N1-H1N...O3	0.90(2)	1.83(2)	2.7185(18)	173(2)
C2-H2A...O3#5	0.99(2)	2.45(2)	3.434(2)	175.4(17)
C2-H2B...O3#6	0.96(2)	2.47(2)	3.284(2)	143.0(17)
N3-H3NA...O2#7	0.77(2)	2.20(2)	2.955(2)	165(2)
N3-H3NB...O4	0.85(2)	2.13(2)	2.976(2)	174(2)
C4-H4A...O1#7	0.96	2.73	3.529(3)	141.6
C4-H4B...O2#7	0.96	2.70	3.568(2)	151.0
O4W-H4WB...O3W#8	0.73(3)	2.10(3)	2.8229(18)	171(3)
O4W-H4WA...O4#3	0.79(3)	2.12(3)	2.903(2)	175(2)

Symmetry transformations used to generate equivalent atoms:

#1 -x+2,-y+1,-z; #2 -x+2,-y+1,-z+1; #3 -x+1,-y+1,-z; #4 x+1,y,z; #5 x-1/2,-y+3/2,z+1/2;
#6 x-1,y,z; #7 x,y,z+1; #8 x-1,y,z-1

Tables for 100K structure

Table S7. Crystal data and structure refinement for jv_zncu_100k_a.

Identification code	jv_zncu_100k_a	
Empirical formula	C8 H32 Cu0.04 N6 O18 S2 Zn0.96	
Formula weight	629.81	
Temperature	100(2) K	
Wavelength	0.7288 Å	
Crystal system	Monoclinic	
Space group	$P2_1/n$	
Unit cell dimensions	a = 6.3912(4) Å	α= 90°.
	b = 27.8593(18) Å	β= 111.237(2)°.
	c = 7.0916(5) Å	γ = 90°.
Volume	1176.94(14) $Å^3$	
Z	2	
Density (calculated)	1.777 Mg/m^3	
Absorption coefficient	1.398 mm^{-1}	
F(000)	656	
Crystal size	0.300 x 0.250 x 0.200 mm^3	
Theta range for data collection	2.999 to 37.434°.	
Index ranges	-10<=h<=10, -46<=k<=46, -11<=l<=11	
Reflections collected	49762	
Independent reflections	5714 [R(int) = 0.0549]	
Completeness to theta = 25.930∞	99.8 %	
Absorption correction	Semi-empirical from equivalents	
Max. and min. transmission	0.767 and 0.675	
Refinement method	Full-matrix least-squares on F^2	
Data / restraints / parameters	5714 / 0 / 217	
Goodness-of-fit on F^2	1.074	
Final R indices [I>2sigma(I)]	R1 = 0.0256, wR2 = 0.0665	
R indices (all data)	R1 = 0.0290, wR2 = 0.0681	
Extinction coefficient	0.0140(12)	
Largest diff. peak and hole	0.516 and -0.467 e. $Å^{-3}$	

Table S8. Fractional atomic coordinates (x 10^4) and equivalent isotropic displacement parameters ($Å^2$x 10^3) for jv_zncu_100k_a. U(eq) is defined as one third of the trace of the orthogonalized U^{ij} tensor.

	x	y	z	U(eq)
Zn1	10000	5000	0	7(1)
Cu1	10000	5000	0	7(1)
O1W	6938(1)	5324(1)	-648(1)	12(1)
O2W	9417(1)	4551(1)	2092(1)	11(1)
O3W	11661(1)	5516(1)	2272(1)	9(1)
S1	6855(1)	6157(1)	3287(1)	7(1)
O2	5230(1)	5897(1)	1511(1)	10(1)
O3	6081(1)	6651(1)	3306(1)	12(1)
O4	6868(1)	5900(1)	5136(1)	12(1)
O5	9087(1)	6149(1)	3167(1)	15(1)
N1	2954(1)	6871(1)	4911(1)	9(1)
C1	2978(1)	6664(1)	6656(1)	9(1)
N2	1543(1)	6880(1)	7338(1)	11(1)
C2	433(2)	7274(1)	5994(1)	11(1)
C3	1488(1)	7252(1)	4401(1)	11(1)
O1	1162(1)	7516(1)	2965(1)	18(1)
N3	4283(1)	6298(1)	7488(1)	12(1)
C4	931(2)	6730(1)	9030(1)	15(1)
O4W	3286(1)	5080(1)	-3953(1)	10(1)

Table S9. Bond lengths [Å] and angles [°] for jv_zncu_100k_a.

Zn1-O1W	2.0502(6)
Zn1-O1W#1	2.0503(6)
Zn1-O2W#1	2.0750(6)
Zn1-O2W	2.0751(6)
Zn1-O3W#1	2.1330(6)
Zn1-O3W	2.1330(6)
O1W-H1WA	0.846(18)
O1W-H1WB	0.814(19)
O2W-H2WA	0.826(18)
O2W-H2WB	0.831(19)
O3W-H3WA	0.852(17)
O3W-H3WB	0.84(2)
S1-O5	1.4594(7)
S1-O3	1.4663(7)
S1-O4	1.4907(7)
S1-O2	1.4973(6)
N1-C1	1.3601(11)
N1-C3	1.3738(11)
N1-H1N	0.894(16)
C1-N3	1.3140(11)
C1-N2	1.3262(11)
N2-C4	1.4526(12)
N2-C2	1.4572(11)
C2-C3	1.5123(13)
C2-H2A	0.958(14)
C2-H2B	0.961(15)
C3-O1	1.2121(11)
N3-H3NA	0.824(16)
N3-H3NB	0.865(17)
C4-H4A	0.9800
C4-H4B	0.9800
C4-H4C	0.9800
O4W-H4WA	0.760(19)
O4W-H4WB	0.803(19)

O1W-Zn1-O1W#1	180.0
O1W-Zn1-O2W#1	88.78(3)
O1W#1-Zn1-O2W#1	91.22(3)
O1W-Zn1-O2W	91.22(3)
O1W#1-Zn1-O2W	88.78(3)
O2W#1-Zn1-O2W	180.0
O1W-Zn1-O3W#1	87.35(3)
O1W#1-Zn1-O3W#1	92.65(3)
O2W#1-Zn1-O3W#1	92.03(3)
O2W-Zn1-O3W#1	87.97(3)
O1W-Zn1-O3W	92.65(3)
O1W#1-Zn1-O3W	87.35(3)
O2W#1-Zn1-O3W	87.97(3)
O2W-Zn1-O3W	92.03(3)
O3W#1-Zn1-O3W	180.00(3)
Zn1-O1W-H1WA	127.5(12)
Zn1-O1W-H1WB	118.9(13)
H1WA-O1W-H1WB	110.9(17)
Zn1-O2W-H2WA	119.4(11)
Zn1-O2W-H2WB	115.6(13)
H2WA-O2W-H2WB	107.3(17)
Zn1-O3W-H3WA	115.7(11)
Zn1-O3W-H3WB	114.5(13)
H3WA-O3W-H3WB	106.4(17)
O5-S1-O3	110.78(4)
O5-S1-O4	110.50(4)
O3-S1-O4	109.64(4)
O5-S1-O2	109.80(4)
O3-S1-O2	109.21(4)
O4-S1-O2	106.83(4)
C1-N1-C3	110.40(7)
C1-N1-H1N	124.1(11)
C3-N1-H1N	125.1(11)
N3-C1-N2	126.80(8)
N3-C1-N1	122.23(8)

N2-C1-N1	110.97(7)
C1-N2-C4	126.79(7)
C1-N2-C2	109.91(7)
C4-N2-C2	122.99(7)
N2-C2-C3	102.38(7)
N2-C2-H2A	112.3(9)
C3-C2-H2A	110.1(9)
N2-C2-H2B	110.0(9)
C3-C2-H2B	111.5(9)
H2A-C2-H2B	110.4(12)
O1-C3-N1	125.70(8)
O1-C3-C2	127.98(8)
N1-C3-C2	106.32(7)
C1-N3-H3NA	119.6(11)
C1-N3-H3NB	119.9(11)
H3NA-N3-H3NB	120.3(15)
N2-C4-H4A	109.5
N2-C4-H4B	109.5
H4A-C4-H4B	109.5
N2-C4-H4C	109.5
H4A-C4-H4C	109.5
H4B-C4-H4C	109.5
H4WA-O4W-H4WB	103.5(18)

Symmetry transformations used to generate equivalent atoms:

#1 -x+2,-y+1,-z

Table S10. Anisotropic displacement parameters ($Å^2$x 10^3) for jv_zncu_100k_a. The anisotropic displacement factor exponent takes the form: $-2\pi^2[h^2 a^{*2}U^{11} + ... + 2 h k a^* b^* U^{12}]$

	U^{11}	U^{22}	U^{33}	U^{23}	U^{13}	U^{12}
Zn1	6(1)	8(1)	6(1)	0(1)	2(1)	0(1)
Cu1	6(1)	8(1)	6(1)	0(1)	2(1)	0(1)
O1W	8(1)	16(1)	10(1)	-4(1)	2(1)	3(1)
O2W	10(1)	13(1)	9(1)	2(1)	4(1)	1(1)
O3W	9(1)	10(1)	10(1)	-1(1)	4(1)	0(1)
S1	7(1)	7(1)	8(1)	-1(1)	3(1)	0(1)
O2	9(1)	11(1)	8(1)	-2(1)	2(1)	-1(1)
O3	15(1)	7(1)	17(1)	0(1)	9(1)	2(1)
O4	15(1)	12(1)	8(1)	2(1)	4(1)	3(1)
O5	8(1)	16(1)	22(1)	-6(1)	8(1)	-1(1)
N1	10(1)	9(1)	9(1)	1(1)	4(1)	1(1)
C1	9(1)	8(1)	9(1)	-1(1)	3(1)	0(1)
N2	11(1)	11(1)	11(1)	1(1)	5(1)	3(1)
C2	11(1)	10(1)	12(1)	0(1)	4(1)	3(1)
C3	12(1)	10(1)	11(1)	0(1)	3(1)	1(1)
O1	24(1)	16(1)	15(1)	7(1)	7(1)	7(1)
N3	15(1)	11(1)	12(1)	3(1)	7(1)	5(1)
C4	17(1)	17(1)	14(1)	1(1)	10(1)	2(1)
O4W	12(1)	11(1)	9(1)	0(1)	3(1)	1(1)

Table S11. Fractional hydrogen coordinates (x 10^4) and isotropic displacement parameters ($Å^2$x 10^3) for jv_zncu_100k_a.

	x	y	z	U(eq)
H1WA	6480(30)	5478(6)	150(30)	30(4)
H1WB	5890(30)	5237(7)	-1640(30)	35(5)
H2WA	10480(30)	4402(6)	2910(30)	26(4)
H2WB	8690(30)	4671(7)	2730(30)	39(5)
H3WA	10810(30)	5724(6)	2520(20)	25(4)
H3WB	12690(30)	5670(7)	2060(30)	40(5)
H1N	3890(30)	6791(6)	4280(30)	27(4)
H2A	730(20)	7579(5)	6660(20)	16(3)
H2B	-1160(30)	7217(5)	5420(20)	20(4)
H3NA	4350(30)	6200(5)	8600(20)	20(4)
H3NB	5160(30)	6181(6)	6920(30)	28(4)
H4A	940	7009	9876	61(6)
H4B	2014	6492	9837	69(7)
H4C	-574	6588	8519	56(6)
H4WA	2980(30)	5206(7)	-4970(30)	33(5)
H4WB	3340(30)	4802(7)	-4230(30)	28(4)

Table S12. Hydrogen bonds for jv_zncu_100k_a [Å and °]. The criteria used are those of the program PLATON[1,2].

D-H...A	d(D-H)	d(H...A)	d(D...A)	<(DHA)
O1W-H1WA...O2	0.846(18)	1.864(18)	2.7004(9)	169.3(17)
O1W-H1WB...O4W	0.814(19)	1.916(19)	2.7274(10)	175.1(19)
O2W-H2WA...O4#2	0.826(18)	1.951(18)	2.7751(9)	175.2(16)
O2W-H2WB...O4W#3	0.831(19)	1.899(19)	2.7263(9)	173.3(19)
O3W-H3WA...O5	0.852(17)	1.789(17)	2.6407(9)	176.9(16)
O3W-H3WB...O2#4	0.84(2)	1.91(2)	2.7410(9)	168.6(19)
N1-H1N...O3	0.894(16)	1.815(16)	2.7039(10)	172.4(16)
C2-H2A...O3#5	0.958(14)	2.412(14)	3.3666(11)	174.1(12)
C2-H2B...O3#6	0.961(15)	2.434(15)	3.2458(11)	142.0(12)
N3-H3NA...O2#7	0.824(16)	2.107(16)	2.9167(10)	167.4(15)
N3-H3NB...O4	0.865(17)	2.098(17)	2.9557(10)	171.3(16)
C4-H4A...O1#7	0.98	2.57	3.5085(12)	160.9
C4-H4B...O2#7	0.98	2.57	3.5399(12)	169.6
O4W-H4WA...O3W#8	0.760(19)	2.024(19)	2.7764(10)	170.7(19)
O4W-H4WB...O4#3	0.803(19)	2.049(19)	2.8482(10)	174.0(17)

Symmetry transformations used to generate equivalent atoms:

#1 -x+2,-y+1,-z; #2 -x+2,-y+1,-z+1; #3 -x+1,-y+1,-z; #4 x+1,y,z; #5 x-1/2,-y+3/2,z+1/2;
#6 x-1,y,z; #7 x,y,z+1; #8 x-1,y,z-1;

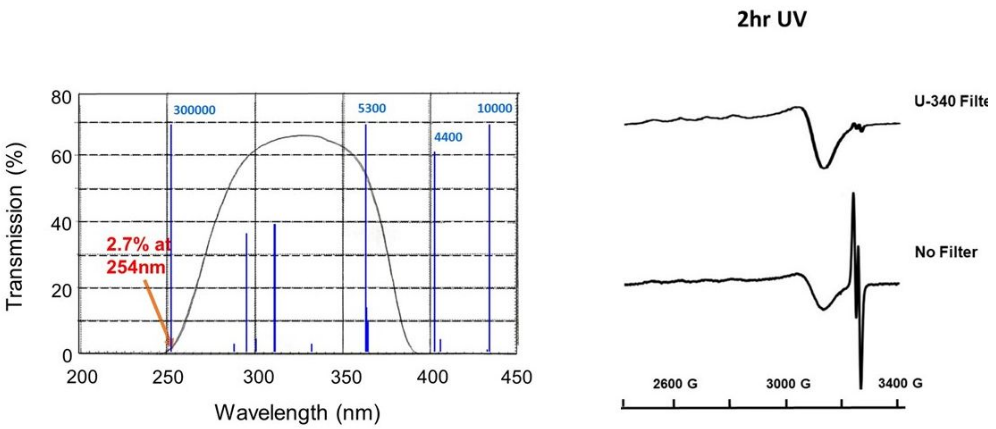

Figure S1. (Left) The Newport U-340 UV filter transmission characteristics measured with a Biomate3 spectrophotometer (black curve) superimposed on emission line intensities (stick numbers are relative, heights not to scale) of the PenRay finger Hg lamp[3]. The measured transmission at 254 nm was 2.7%. (Right) EPR spectra of copper-doped ZnCrnS UV-irradiated for 2hr at room temperature with (top) and without (bottom) passage through the U-340 filter.

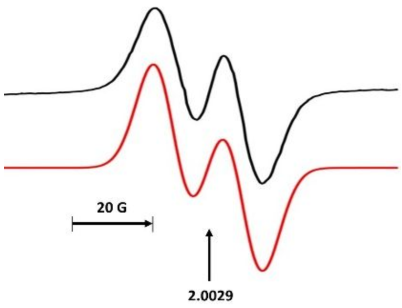

Figure S2. EPR powder spectra of the free radical pattern in room temperature UV irradiated copper-doped Zinc creatninium sulfate. The black curve is the experimental spectra, and the red curve is the simulation-fit using EasySpin. Fit parameters are given in Table 3.

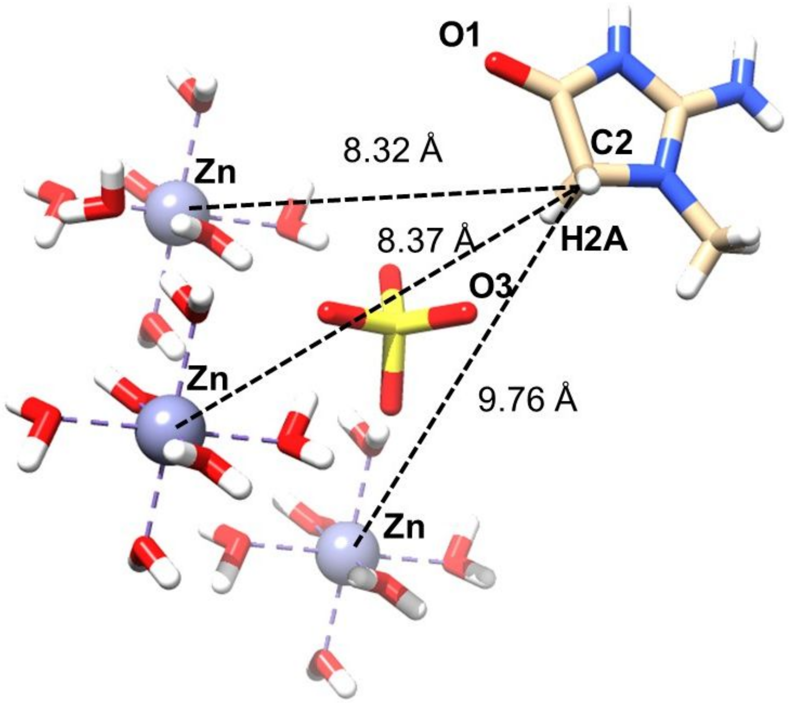

Figure S3. The three nearest metal-hexahydrate sites that interact with the sulfate ion that is postulated to facilitate the excited-state PCET mechanism. This is accomplished by allowing electron transfer from the creatininium through the sulfate to doped Cu(II) and accepting the released H2A proton thus stabilizing the creatinine cation radical. The non-bonded distance (100K structure) from transition ion to the C2 of creatininium for the three sites are labeled.

References

1. Speck, A.L. Structure validation in chemical crystallography. *Acta Cryst.* **2009**, *D65*, 148-155.
2. Speck. A.L. Single crystal structure validation with the program PLATON. *Journal of Applied Crystallography* **2003**, *36*, 7-13.
3. Sansonetti, C.J.; Salit, M.L.; Reader, J. Wavelengths of spectral lines in mercury pencil lamps *Applied Optics* **1996**, *35* (1), 74-77.
